# NHS-Functionalized THP Derivative for Efficient Synthesis of Kit-Based Precursors for ^68^Ga Labeled PET Probes

**DOI:** 10.3390/biomedicines9040367

**Published:** 2021-04-01

**Authors:** Giuseppe Floresta, George P. Keeling, Siham Memdouh, Levente K. Meszaros, Rafael T. M. de Rosales, Vincenzo Abbate

**Affiliations:** 1 Department of Analytical, Environmental and Forensic Sciences, King’s College London, London SE1 9NH, UK; giuseppe.floresta@kcl.ac.uk (G.F.); siham.memdouh@kcl.ac.uk (S.M.); 2 School of Biomedical Engineering & Imaging Sciences, King’s College London, St Thomas’ Hospital, London SE1 7EH, UK; george.keeling@kcl.ac.uk (G.P.K.); leventemeszaros@nano-mab.com (L.K.M.); rafael.torres@kcl.ac.uk (R.T.M.d.R.); 3 NanoMab Technology (UK) Ltd., 720 Centennial Court, Centennial Park, Elstree, Hertfordshire WD6 3SY, UK

**Keywords:** GLP-1, PET imaging, gallium-68, molecular imaging, molecular probe, peptides synthesis, THP, hydroxypyridinone chelators, N-Hydroxysuccinimide (NHS) derivatives, single-domain antibody, sdAb

## Abstract

Hexadentate tris(3,4-hydroxypyridinone) ligands (THP) complex Fe^3+^ at very low iron concentrations and their high affinities for oxophilic trivalent metal ions have led to their development for new applications as bifunctional chelators for the radiometal gallium-68 (^68^Ga). THP-peptide bioconjugates rapidly and quantitatively complex ^68^Ga at room temperature, neutral pH, and micromolar ligand concentrations, making them amenable to kit-based radiosynthesis of ^68^Ga PET radiopharmaceuticals. With the aim to produce an *N*-hydroxysuccinimide-(NHS)-THP reagent for kit-based ^68^Ga-labeling and PET imaging, THP-derivatives were designed and synthesized to exploit the advantages of NHS chemistry for coupling with peptides, proteins, and antibodies. The more stable five-carbon atoms linker product was selected for a proof-of-concept conjugation and radiolabeling study with an anti-programmed death ligand 1 (PD-L1) camelid single domain antibody (sdAb) under mild conditions and further evaluated for site-specific amide bond formation with a synthesized glucagon-like peptide-1 (GLP-1) targeting peptide using solid-phase synthesis. The obtained THP-GLP-1 conjugate was tested for its ^68^Ga chelating ability, demonstrating to be a promising candidate for the detection and monitoring of GLP-1 aberrant malignancies. The obtained sdAb-THP conjugate was radiolabeled with ^68^Ga under mild conditions, providing sufficient labeling yields after 5 min, demonstrating that the novel NHS-THP bifunctional chelator can be widely used to easily conjugate the THP moiety to different targeting molecules (e.g., antibodies, anticalins, or peptides) under mild conditions, paving the way to the synthesis of different imaging probes with all the advantages of THP radiochemistry.

## 1. Introduction

Radiometals are radioactive metal isotopes that can be exploited in medical diagnosis and cancer therapy. To use these isotopes effectively for medical applications, the free radiometal ion—present in low concentrations—must be sequestered from aqueous solution using a chelating agent. To target the radiometal to a specific tissue, a targeting molecule is covalently bonded to the chelating molecule used for this application, making an active radiopharmaceutical as a diagnostic and/or radiotherapeutic agent. When the targeting molecule, engineered with a chelator, is injected into a patient, it should deliver the radioactive isotope without radiometal loss and supply an in vivo site-specific radioactive source for imaging or therapy purpose. A quickly expanding number of radiometals with a broad variety of characteristics (i.e., half-lives, emission types, energies, and branching ratios) are constantly being developed. Thanks to the wide range of generated and available radionuclides, it is currently possible to meticulously pick the specific nuclear properties that are needed for the specific application. ^68^Ga, ^64^Cu, ^86^Y, ^89^Zr, and ^44^Sc are some examples of radiometals that are currently used clinically for positron emission tomography (PET) imaging, providing a non-invasive and sensitive method to obtain quantitative images of many molecular processes and targets in the human body [1].

**Figure 1:**
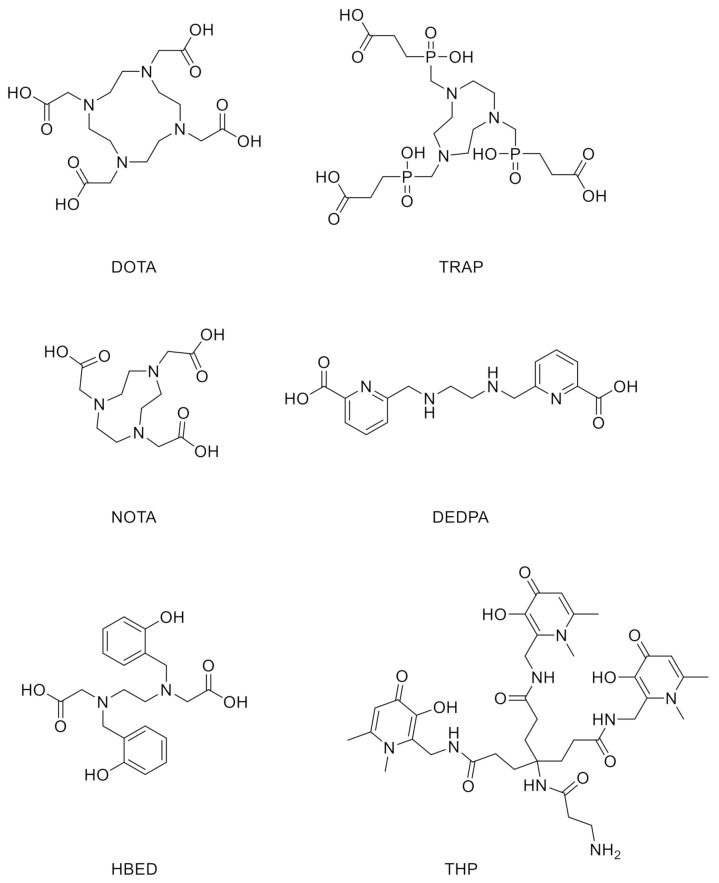
Structures of some ^68^Ga chelators of current generation.

Each radiometal ion has unique coordination chemistry properties in aqueous environments and it is thanks to these different characteristics that they can be safely used for medical applications. The main distinction between the radioactive (hot) and the nonradioactive (cold) metal ion chemistry is that the radiochelation is usually executed under extremely dilute conditions, with metal ions being used in nM to pM concentrations. As a result of these low concentrations, the chelating moiety of the radiopharmaceutical must be coordinated efficiently by the metal in order to become clinically useful. Interestingly, different radiometals used in PET imaging have multiple radioactive isotopes that are useful for diagnostic as well as therapeutic purposes (e.g., ^86/90^Y, ^67/68^Ga, ^44/47^Sc, ^60/61/62/64^Cu), and all isotopes of a given element have identical chemistry. This means that a same radiopharmaceutical agent can easily be labeled with different radioisotopes with therapeutic or diagnostic properties. Radiopharmacies in hospitals have been developed around technetium-99m chemistry and protocols for labeling the tracer. When technetium-99m (^99m^Tc) is used as radioactive source the tracer is labeled with speed, simplicity, and reproducibility from commercially available “cold kits” compatible with good manufacturing practice (GMP) [2]. With the growth of ^18^F and ^11^C-based PET imaging tracers, more complex and costly infrastructures have been built to support PET because a kit model is not compatible with those tracers and due to the need for an on-site cyclotron and more complex synthetic chemistry procedures involved in radiolabeling. Modern ^68^Ga generators are compatible with GMP and have the potential to be used in the manufacturing of kit-based radiotracers if a simple chelation step can be achieved [3,4], making ^68^Ga tracers widely available without the costly infrastructures related with ^18^F and ^11^C production. Despite several efforts for a kit-based ^68^Ga tracer production [5], an ideal one-step procedure for gallium radiolabeling, with the same simplicity of the long-established technetium labeling procedures (addition of generator eluate to the kit vial), has not yet been widely achieved [6,7]. The current generation of ^68^Ga chelators does not satisfy optimal criteria (i.e., the radiolabeling reaches completion quickly, i.e., in a matter of minutes at room temperature, must not be affected by common trace metals, must be ready to use without additional steps to concentrate, buffer or purify, should also resist to the in vivo transchelation and the conjugation and radiolabeling should not produce mixtures of diastereomers, enantiomers, or geometric isomers with potential adverse pharmacokinetics). Some examples of current ^68^Ga chelators are shown in Figure 1. Among them, DOTA [8], which binds gallium with extraordinary stability, has very slow complexing kinetics that requires heating and low pH conditions. Moreover, the low radiochemical yields (<95%) require a purification step. NOTA, TRAP, and DEDPA (Figure 1) are promising chelators, however, similarly to DOTA, their gallium complexation require acidic conditions, and the formed complexes are particularly vulnerable to competition from contaminating trace metals [9,10,11,12]. The DOTA series of chelators show rapid, room temperature labeling at pH 5 but require preprocessed eluate [13]. All of these disadvantages are not compatible with a kit-based protocol and GMP.

A recent class of chelators that promises to have all the requirements for kit-based radiolabeling are based on tris(hydroxypyridinone) (THP) [14]. THP can complex ^68^Ga rapidly at room temperature and pH = 7, with high yield and chromatographically pure compound. Its performance has previously been evaluated against a range of common chelators, showing better properties for radiolabeling under mild conditions. THP has also been functionalized for conjugation to peptides, proteins, and other targeting molecules such as bisphosphonates while retaining the required mild radiolabeling and in vivo targeting properties [15,16,17,18]. Moreover, peptide-THP-based PET tracers have been already tested in clinical trials with promising results [19].

**Figure 2:**
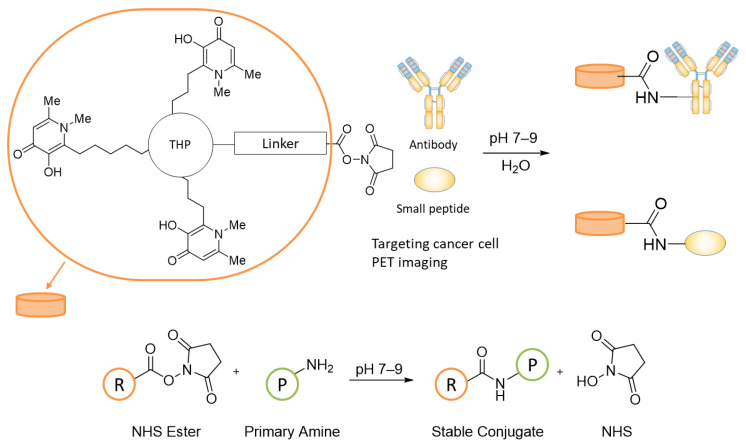
Schematic use of THP-NHS and NHS ester chemical conjugation to an amine. R represents an NHS activated reagent; P represents a protein or other molecules that contain an amino group.

With the aim to exploit the *N*-hydroxysuccinimide (NHS) ester chemistry and to produce an easy to use kit-based NHS-THP for ^68^Ga-labeling and PET imaging, in this study, a “biochemically-friendly” NHS-functionalized THP-derivative was designed and synthesized. This derivative offers the advantage of well-established NHS chemistry for the successful and biocompatible coupling with virtually any protein sequence including antibodies, in a single step reaction in an aqueous environment (Figure 2) [20]. NHS ester-activated compounds react with primary amines under physiological conditions to slightly alkaline conditions (pH 7.2 to 9) to yield stable amide bonds. The reaction only releases NHS and does not have the limitation of the already proposed THP derivatives, namely THP-maleimide and THP-isothiocyanate (i.e., presence of a thiol group, partial reduction of cysteine in antibodies and/or water instability) [16,21,22]. After a biocompatible reaction with the activated NHS-THP, the antibody or protein could directly be used in PET imaging after radiolabeling. With this objective in mind, we designed two different NHS-THP ligands and evaluated their stability and amide bond formation capabilities under either site-selective solid-phase synthesis, or solution-phase under mild conditions. The more stable six-carbon-atom product was selected for a proof of concept conjugation and radiolabeling study with an anti-programmed death ligand 1 (PD-L1) camelid single domain antibody (sdAb) MY-1502-6-51 under mild conditions and further evaluated for its site-selective amide bond formation abilities with a synthesized glucagon-like peptide-1 (GLP-1) targeting peptide using solid-phase synthesis. The THP-GLP-1 targeting peptide obtained was then evaluated for its ^68^Ga chelating ability, demonstrating promising properties for the detection and monitoring of GLP-1 aberrant malignancy.

GLP-1 is a 30-amino acid peptide released in the gut following the ingestion of nutrients. It acts as a hormone by stimulating the insulin release from the β-cells to maintain glucose homeostasis. It also inhibits glucagon production, hepatic gluconeogenesis, gastric mobility, and suppresses appetite [23]. In line with the physiological role of GLP-1, high levels of its target receptor (GLP-1R) are expressed in the pancreatic β-cells. However, the action of GLP-1 is strictly regulated to prevent the development of hypoglycemia by rapid degradation and deactivation of the peptide by the action of an enzyme called dipeptidyl-peptidase-IV (DPP-4) [24,25,26]. GLP-1R has been shown to be expressed in high density and high incidence in some types of cancers derived from endocrine, neuroendocrine, and embryonic origins (e.g., insulinoma) [27,28,29].

**Figure 3:**
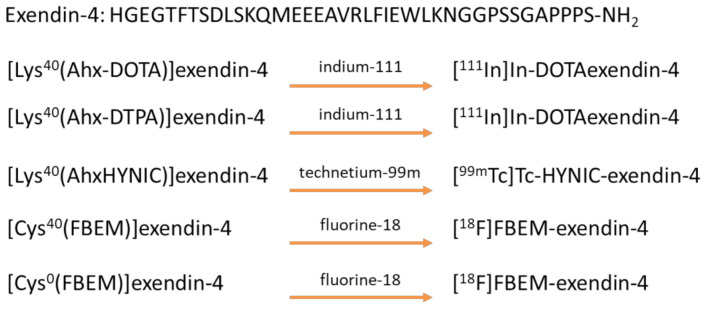
Sequence of extendin-4 and GLP-1-like radiopeptides with high binding affinity to GLP-1R.

Due to its rapid metabolism and inactivation in vivo, a bioconjugate incorporating the original GLP-1 sequence is not suitable to target GLP-1R expression [30]. Interestingly, some drugs that are normally used for the therapy of type 2 diabetes, despite having a different peptide structure, retain an affinity to the GLP-1R but they are not metabolized by the DPP-4 [31]. One example is a linear 39 amino acid peptide named Exendin-4 (Figure 3) [32]. There are several published examples (Figure 3) of a modified version of the exendin-4 adapted for the use in PET imaging. Some examples are listed in Figure 3 ([Lys^40^(Ahx-DOTA)]exendin-4 [33], [Lys^40^(Ahx-DTPA)]exendin-4, [34] [Lys^40^(AhxHYNIC)]exendin-4 [35,36], [Cys^40^(FBEM)]exendin-4 [37], [Cys^0^(FBEM)]exendin-4 [37]). In these examples, the primary sequence of the exendin-4 was in all cases extended at the carboxylic or the amino terminal groups with the added amino acid possessing the right characteristic for rendering it a radiopharmaceutical agent [38].

The sequence of Exendin-4 was chosen as GLP-1 targeting peptide in this study after the modification with the newly designed NHS-THP.

sdAbs are natural antibody fragments with a molecular weight of approximately 15 kDa, derived from a class of camelid antibodies expressed by camels and llamas. Due to their high antigen-binding affinity, stability, and rapid blood clearance, sdAbs are increasingly used as diagnostic and therapeutic tracers in nuclear medicine [39,40,41]. The programmed cell death 1 (PD-1)/PD-L1 immune checkpoint has a key role in the immune evasion of cancer cells in several malignancies, such as non-small cell lung cancer. Several antibodies targeting this immune checkpoint have been granted regulatory approval in recent years and there is an increasing need for imaging agents for the diagnosis and monitoring of PD-(L)1 positive malignancies. Several sdAbs targeting cancer biomarkers such as PD-L1 or human epidermal growth factor receptor 2 are currently in clinical development [40,41,42].

## 2. Experimental Section

### 2.1. Synthesis

#### 2.1.1. Materials for the Synthesis

Materials and chemicals were purchased from Acros Organics (Thermo Fisher Scientific, Waltham, MA, USA) and Merck KGaA (St. Louis, MO, USA) and were reagent grade or better. The anti-PD-L1 sdAb MY-1502-6-51 was provided by NanoMab Technology Ltd. (Centennial Park, Elstree, Borehamwood, Hertfordshire, UK). Solvents and NMR solvents were purchased from Fisher Scientific (Thermo Fisher Scientific, Waltham, MA, USA) and, Merck KGaA, and VWR (Radnor, PA, USA). Silica gel for column chromatography was purchased from Merck. All samples were dried in a vacuum oven connected to a vacuum pump (BOC-Edwards.). Silica gel 60 F254, Merck pre-coated aluminum sheets were employed for thin-layer chromatography (TLC) and spots were visualized under UV light. ^1^H NMR and ^13^C NMR spectra were recorded on A Bruker Avance III HD NanoBay 400 MHz NMR with a 5 mm ^1^H/^13^C/^15^N/^31^P QNP probe equipped with z-gradient. Tetramethylsilane (TMS) was used in all NMR experiments as internal standard and chemical shift (*δ*) values are given in ppm. General NMR Analysis Toolbox (GNAT, v1.2, Free Software Foundation, Inc., Boston, MA, USA) was used for the analyses of NMR experiments [43]. Low resolution mass spectra were obtained on a Thermofisher LCQ DECA XP ion trap mass spectrometer or a Waters-Micromass ZQ-Single quadrupole mass spectrometer. The GLP-1-THP derivative **8** and the sdAb and its conjugates were analyzed via high-resolution-MS (direct infusion) on a Thermo Exactive Orbitrap MS operating in ESI^+^ mode. Isotopic distributions were calculated using Molecular Weight Calculator version 6.46. The resulting graph data were extracted and processed in Microsoft Excel. Analytical RP-HPLC was carried out on a HP1050 HPLC system equipped with an autosampler, a quaternary pump and a diode-array detector (DAD). The HPLC column was a Zorbax SB C-18 2.1 mm × 10 cm (particle size 5 micron) The employed flow rate was 0.3 mL/min and the eluents were monitored at wavelengths between 210–280 nm. A linear gradient of mobile phase B (acetonitrile (ACN) containing 0.1% trifluoroacetic acid (TFA)) over mobile phase A (0.1% TFA in water) from 0–90% B in 20 min was performed. Data were collected and analyzed using ChemStation software. The concentration of sdAb aliquots was determined by UV spectroscopy at a detection wavelength of 280 nm in a Thermo Fisher NanoDrop UV spectrophotometer based on the 25,690 M^−1^ cm^−1^ calculated extinction coefficient of MY-1502-6-51.

#### 2.1.2. Synthesis of THP (5)

Compound **2** was prepared according to an already reported procedure [44]. The lyophilized compound **2** (1 mmol) was dissolved in anhydrous dichloromethane (DCM) (1 mL) and chilled in an ice bath for 10 min. An excess of BCl_3_ (5 mL of a solution 1 M in DCM) was added and the solution was stirred over a stream of nitrogen. The resulting mixture was allowed to slowly warm to room temperature over nitrogen and then stirred overnight. The solution was again chilled in an ice bath and methanol (50 mL) was then added to quench the reaction. After the methanol addition, the reaction was further stirred for 1 h. The resulting solution was evaporated under reduced pressure, dried under high vacuum for 3 h, dissolved in a minimal amount of methanol and then precipitated in ice-cold diethyl ether. Following centrifugation, the pellet was washed several times with diethyl ether and finally dissolved in 50% ACN in water containing 0.1% TFA and freeze-dried for 24 h. The freeze-dried compound **5** was used without further purification. Yield: 99%. ^1^H NMR (400 MHz, DMSO (Dimethyl Sulfoxide)-d6) δ 1.81 (t, J = 8.6 Hz, 2H), 2.12 (dd, J = 11.3, 5.1 Hz, 2H), 2.66–2.42 (m, 5H), 2.98–2.83 (m, 1H), 3.90 (s, 3H), 4.57 (d, J = 5.1 Hz, 2H), 7.33 (d, J = 4.0 Hz, 1H), 8.09 (s, 1H), 9.02 (s, 1H). ^13^C NMR (101 MHz, DMSO) δ 21.11, 29.79, 30.51, 32.99, 35.24, 35.65, 39.34, 39.54, 39.75, 39.96, 40.17, 40.38, 40.59, 57.61, 113.23, 140.55, 143.10, 149.07, 159.92, 169.64, 173.89. Calculated ESI^+^ [M + H]^+^: 769.4. Measured ESI^+^ [M + H]^+^: 769.4 (Figures S1–S3).

#### 2.1.3. General Procedure for the Synthesis of bis-NHS-Succinnic/Glutaric Acid Ester (3,4)

To a 0 °C cooled solution of N-hydroxysuccinimide (27.7 mmol) in dry tetrahydrofuran (10 mL), was added triethylamine (27.7 mmol). The appropriate acid dichloride (succinyl chloride **3**, glutaryl chloride **4**) (12.6 mmol) was then added in a dropwise fashion over 10 min. The resulting white suspension was stirred for 2 h at room temperature, followed by solvent evaporation under vacuum. The residue was taken up in DCM (100 mL) and washed with water (3 × 50 mL). Drying over MgSO_4_, filtration and evaporation of the solvent yielded a white solid (**3** 95%, **4** 90%), which was recrystallized from isopropyl alcohol [45].

#### 2.1.4. General Procedure for the Synthesis of THP-Succinic/Glutaric Acid Ester (6,7)

*N*,*N*-Diisopropylethylamine (DIPEA) (6 equiv, 6 mmol) was added to compound **5** (1 mmol) in 1 mL of dimethylformamide (DMF) and then this solution was added dropwise into a solution of bis-NHS acid ester derivative (**3** or **4**) (10 mmol) in DMF (5 mL) at 0 °C. The reaction was monitored by MS direct infusion and HPLC-UV. All the reactions were completed within 3 h. Reactions were dried under vacuum and chromatographed via preparative HPLC/UV using H_2_O/0.1% TFA as eluent A and ACN/0.1% TFA as eluent B. The elution program used a linear gradient of 0%–60% of eluent B in 60 min. The detection wavelength was 281 nm and the flow rate was 15 mL/min. Only the hydrolyzed product was then isolated for molecule **6**. THP-glutaric **7**; Yield: 85%; ^1^H NMR (400 MHz, D_2_O) δ 1.72–2.82 (m, 10H), 2.04–2.12 (m, 10H), 2.30–2.43 (m, 2H), 2.65 (s, 2H), 3.73 (s, 2H), 3.77 (s, 9H), 4.53 (s, 6H), 6.88 (s, 3H). Calculated ESI^+^ [M + H]^+^: 980.4. Measured ESI^+^ [M + H]^+^: 980.4 (Figures S4 and S5).

#### 2.1.5. Synthesis of GLP-1-Peptide-THP (8)

The precursor GLP-1-peptide-THP linear sequence was synthesized using standard Fmoc solid-phase peptide synthesis approach. All the Fmoc-(fluorenylmethyloxycarbonyl)-amino acid residues contained in the sequence were side-chain protected with classical acid-labile protecting groups except from the first Lys attached to the resin that was protected with -Dde. Rink amide resin (ProTide™, loading 0.55–0.8 mmol/g, 0.4 g) was used as solid support. For the loading of the resin 3 equiv. (equivalents) of Fmoc-protected amino acid Lys(Dde ((1-(4,4-Dimethyl-2,6-dioxocyclohex-1-ylidene)-3-ethyl)), 3 equiv. of oxyma and 3 equiv. of DIC (*N*,*N*′-Diisopropylcarbodiimide) were dissolved in DMF (2 mL) and the reaction mixture was added to the resin. The reaction mixture was allowed to mix on a tube roller mixer overnight at room temperature. The resin was then washed three times with DCM (20 mL) and DMF (20 mL). For Fmoc-deprotection, the resin was treated two times for 15 min. with 20% piperidine/DMF (10 mL).

A standard protocol was then used for the peptide elongation. In each step, the resin was reacted with 4 equiv. of Fmoc-protected amino acid, 4 equiv. of oxyma, and 4 equiv. of DIC in DMF (2 mL). The reaction mixture was allowed to mix on a tube roller mixer for 3 h at room temperature. Fmoc-deprotections were performed with 20% piperidine/DMF (10 mL). At the end of the sequence, the last amino acid (Serine) was deprotected and acetylated by reaction with 10 equiv. of acetic anhydride and 20 equiv. of DIPEA in a solution of DMF (3 mL) and then added to the resin and shaken for 30 min.

After the removal of the Dde group from the lysine in presence of a solution of 2% hydrazine in DMF (10 mL), the NHS-THP (**7**) was site-specifically (^40^Lys) coupled under mild conditions using 5 equiv. of **7** in DMF by shaking the solution for 12 h without the use of activation agents. After the coupling of the hexadentate tris(3,4-hydroxypyridinone) **7**, the peptide was removed from the resin.

For the cleavage of the peptide from the solid phase, the resin was treated two times for 15 min. with a solution of TFA/TIPS (triisopropylsilane)/thioanisole/water (97/1/1/1, 10 mL). The solution was then concentrated, and the crude product was isolated by precipitation into cold diethyl ether. The precipitate was collected by centrifugation and dried under vacuum. The crude product was analyzed by RP-HPLC and mass spectrometry. The final compound was chromatographed via preparative HPLC/UV using 0.1% TFA in H_2_O as eluent A and 0.1% TFA in ACN as eluent B. The elution program used a linear gradient of 0%–40% of the eluent B in 60 min. The detection wavelength was 281 nm, and the flow rate was 15 mL/min. The isolated fractions were further analyzed by analytical HPLC-DAD, using the same above-mentioned mobile phase of the preparative purification. The elution program used a linear gradient of 0%–95% of eluent B in 20 min using a C18 column at a 0.3 mL/min flow rate. Calculated ESI^+^ [M + 4H]^4+^: 1295.8860, [M + 5H]^5+^: 1036.9102, [M + 6H]^6+^: 864.2597, [M + 7H]^7+^: 740.9380. Measured ESI^+^ [M + 4H]^4+^: 1295.6461, M + 5H]^5+^: 1036.9208, [M + 6H]^6+^: 864.1021, [M + 7H]^7+^: 740.8036.

#### 2.1.6. Synthesis of MY-1502-6-51-THP

In this preliminary set of experiments, MY-1502-6-51-THP (**10**) was synthesized in 20-fold and 40-fold molar excess of **7** as follows. A 50 mg/mL stock solution of **7** in anhydrous DMSO was prepared and used immediately after preparation. To a 200 µL aliquot of 2 mg/mL sdAb in PBS (pH 7.4), 1.03 µL (20-fold molar excess) or 2.06 µL (40-fold molar excess) of 50 mg/mL **7** in DMSO were added, the reaction mixtures were incubated at room temperature for 5 h. After incubation, excess **7** was removed by 6 times repeated ultrafiltration, using centrifugal filter units with a molecular weight cut-off of 10 kDa (Amicon, Merck Millipore). The sdAb-THP conjugate was recovered in PBS in a 1.7 mg/mL solution after the last filtration step and stored at 2–8 °C until radiolabeling.

To confirm that the binding of ^68^Ga to the MY-1502-6-51, sdAb was THP-mediated, a control sample was prepared in the presence of **5**, a non-functionalized THP derivative that is not expected to covalently bind to the sdAb sequence. Briefly, a 230 µl aliquot of MY-1502-6-51 in PBS (pH 7.4), corresponding to 480 µg of protein was mixed with 11.5 µL of a 48 mg/mL solution of **5** in DMSO, corresponding to an 18-fold molar excess of **5**. The mixture was incubated at room temperature for 5 h. Excess **5** was removed by ultrafiltration repeated six times, using centrifugal filter units with a molecular weight cut-off of 10 kDa (Amicon, Merck Millipore). The sdAb was recovered in PBS in a 2.2 mg/mL solution after the last filtration step and stored at 2–8 °C until radiolabeling.

Calculated monoisotopic mass of [MY-1502-6-51]: 15178.3; accurate mass for [M + 10H]^10+^ for [MY-1502-6-51] 1518.1 (exact monoisotopic mass = 1518.8), and accurate mass for [M + 10H]^10+^ for [10]: 1603.1 (exact monoisotopic mass = 1603.8).

### 2.2. Radiochemistry

#### 2.2.1. Materials for ^68^Ga Radiolabeling of GLP-1-Peptide-THP

Gallium-68 was eluted from an Eckert & Ziegler (E&Z Radiopharma GmbH, Berlin, Germany) ^68^Ge/^68^Ga generator producing 200–400 MBq of [^68^Ga]GaCl_3_, using hydrochloric acid (5 mL, 0.1 M) and collected in five different fractions (1 mL). Capintec CRC-25R was used as radiation counter. Radioactive HPLC was performed on an Agilent Technologies 1260 Infinity system equipped with degasser, UV detector (220 nm); radioactive detection was done by a Bioscan Inc. B-FC-3200 photomultiplier tube (PMT) detector. Laura software (LabLogic Systems Ltd., Sheffield, UK) was used to collect and analyze all radio HPLC data. The reverse-phase method used is shown in Method 1 (Table 1).

An Agilent Eclipse XDB-C18 column with 5 μm particle size and column dimensions of 4.6 × 150 mm for the analytical reverse-phase HPLC. Radio instant thin layer chromatography (ITLC) was developed on Agilent Technologies glass microfiber chromatography paper impregnated with silica gel (ITLC-SG) and analyzed using a Lablogic Flow-count TLC scanner and a BioScan B-FC-3200 PMT detector using Laura software. Two different ITLC methods were used: (1) the acetate method (1 M ammonium acetate in water/methanol (1:1)); (2) citrate method (0.175 M citric acid, 0.325 M trisodium citrate in water).

#### 2.2.2. Sample Preparation for ^68^Ga Radiolabeling of GLP-1-Peptide-THP

Stock solutions of two GLP-1-peptide-THP (**8**) samples in water were prepared at a concentration of 5 mg mL^−1^ (836 μM) using 0.8 and 0.45 mg of sample respectively. Stock solutions were stored at −20 °C when not in use.

#### 2.2.3. GLP-1-Peptide-THP Radiolabeling

5 μL (4.26 nmol) of the stock solution of GLP-1-peptide-THP (**8**) were added to [^68^Ga]GaCl_3_ (250 μL, 5–90 MBq). Sodium bicarbonate solution in water (26 μL, 1 M) was added immediately after. The mixture was stirred, and the pH was checked to ensure it was in the range 6.5–7.5. Radiochemical yield and purity was evaluated after 5 min using ITLC by both the acetate method (^68^Ga Rf = 0, [^68^Ga]Ga-THP-GLP-1 Rf = 0) and citrate method (^68^Ga Rf = 0.8–1, [^68^Ga]Ga-THP-GLP-1 Rf = 0) and after 10 min by HPLC (Reverse phase: unbound ^68^Ga retention time (Rt) = 1.9 min, [^68^Ga]Ga-THP-GLP-1 Rt = 13.7–13.9 min.

#### 2.2.4. Materials for ^68^Ga Radiolabeling of the sdAb-THP Conjugate MY-1502-6-51-THP

Gallium-68 chloride was eluted from an Eckert & Ziegler (E&Z Radiopharma GmbH, Berlin, Germany) ^68^Ge/^68^Ga generator in 0.1 M HCl. HPLC analyses were performed on an Agilent Technologies 1200 HPLC system equipped with a variable wavelength detector for UV detection at 280 nm and coupled to a Phenomenex BioSep s2000 size exclusion chromatography column (7.8 × 300 mm, 5 μm particle size) (Phenomenex, Torrance, California, USA) and connected to a Bioscan Inc. B-FC-3200 PMT detector (Bioscan Inc., Washington, DC, USA). Samples were eluted over 30 min at a flow rate of 1 mL/min in 45% ACN and 0.1% TFA in water (Method 2 for HPLC).

#### 2.2.5. MY-1502-6-51-THP Radiolabeling

A mixture of 30 μg (1.9 nmol) **10** in 18 μL PBS and 9 μL 1 M NaHCO_3_ in water was incubated with 100 μL [^68^Ga]GaCl_3_ (ca. 15 MBq) in 0.1 M HCl for 5 min at room temperature, the pH of the reaction mixture was around 5. Samples were analyzed by HPLC (Method 2), ^68^Ga-MY-1502-6-51-THP (**11**) Rt = 6.7–6.8 min, unbound ^68^Ga eluted in a broad peak Rt = 10.7–10.8 min. **11** was identified based on the UV signal corresponding to **10** (Rt = 6.2–6.4 min). To confirm that the formation of **11** was THP-mediated, the same sdAb incubated with THP(**5**) was radiolabeled as follows: a mixture of 44 μg MY-1502-6-51 in 22 μL PBS and 19.5 μL 1 M NaHCO_3_ in water were incubated with 200 μL [^68^Ga]GaCl_3_ (ca. 33 MBq) in 0.1 M HCl for 5 min; the pH of the reaction mixture was around 5. Samples were analyzed by HPLC (Method 2).

## 3. Results and Discussion

### 3.1. Design and Synthesis of NHS–THP

**Figure 4:**
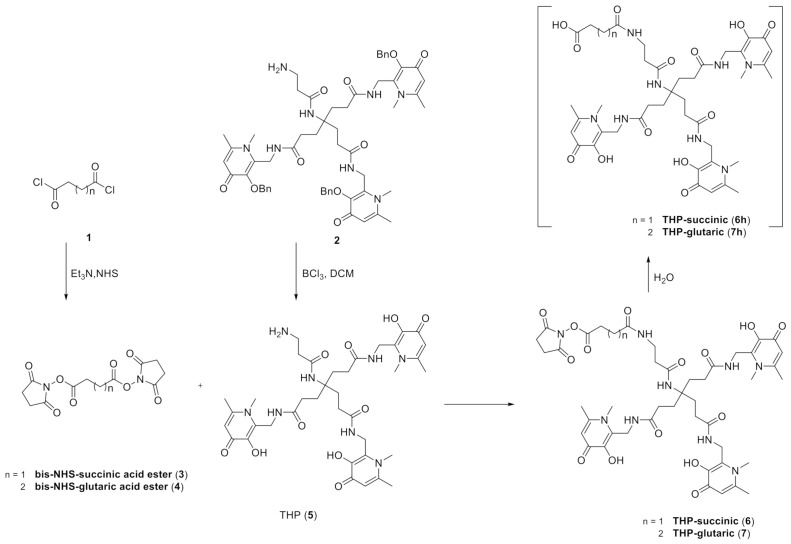
Synthetic scheme for THP-succinic (**6**) and THP-glutaric (**7**) and their hydrolysis products **6h** and **6h**.

With the aim to produce a kit-based NHS-THP for effortless and efficient gallium labeling under physiological conditions, two THP-derivatives (**6**,**7**) were designed and synthesized for exploiting the advantages of NHS chemistry for the successful and biocompatible coupling with antibodies and proteins [20]. With this objective in mind, we started with the synthesis of hexadentate tris(3,4-hydroxypyridinone) (**2**) (Figure 4) as already reported [44]. Once the synthesis of molecule **2** was completed, the compound was first deprotected with BCl_3_ and then conjugated to two different linkers with 4- and 5- atoms. Two bis-NHS-acid esters were used as linkers: bis-NHS-succinic acid ester (**3**) and bis-NHS-glutaric acid ester (**4**) synthesized as previously reported in literature [45] (Figure 4), by the reaction of the respective acyl chloride (**1**) with NHS. Treatment of **5** with 10 equiv. of the respective bis-NHS acid ester derivative in DMF produced the activated ester: THP-succinic (**6**) and glutaric (**7**). The reaction was monitored by ESI^+^ direct infusion MS and HPLC with UV detection. The results of the monitoring were the following: for compound **6** both direct infusion mass spectra and HPLC-DAD revealed a major presence of the hydrolyzed acid derivative **6h**, with only a small part of unhydrolyzed THP-succinic (**6**). Conversely, for the other compound, **7** (THP-glutaric), the outcome was different, and only the mass of the final molecule **7** (non-hydrolyzed NHS ester) was detected by direct infusion MS, and just a small portion of hydrolyzed compounds **7h** was detected by HPLC/UV chromatogram for the glutaric derivative.

**Figure 5:**
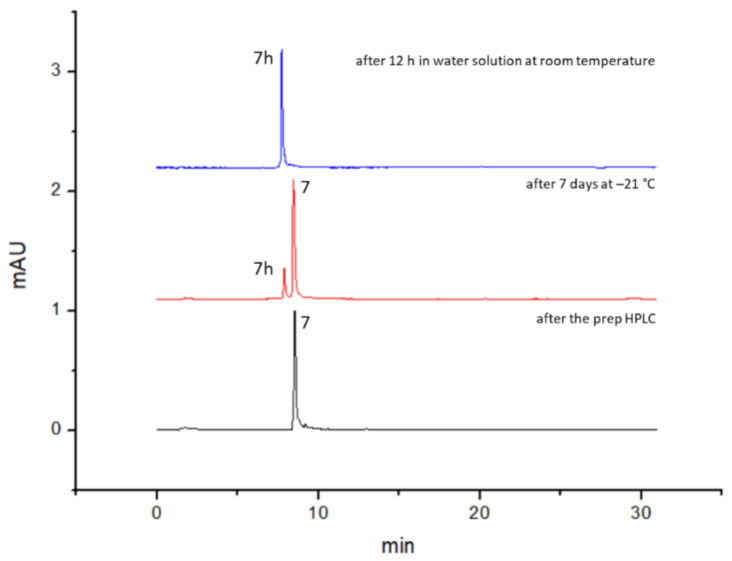
THP-glutaric (**7**) preliminary stability tests. Black line, after the preparative HPLC. Red line, after 7 days storage at −21 °C, dried compounds. Blue line, after 12 h incubation in water solution at room temperature.

We hypothesized that in both cases the THP-NHS ester derivatives **6-7** were generated. However, these compounds are very reactive under the conditions of the HPLC analysis (dilute acidic solution), and the succinic derivative (**6**) decomposed instantaneously after the addition of acidic water for the mass spectrometry analysis, probably because of its unique planar structure with a partially non-saturated C–C bonds of its molecular skeletons that led to this different reactivity [46]. Considering the obtained results, only the THP-glutaric (**7**) was purified by preparative HPLC and analyzed for its long-term stability. The compound was then purified via preparative HPLC/UV as described in the experimental section. The major purified product was the THP-NHS ester derivative **7** but the acid derivative (hydrolyzed product, compound **7h**) was also collected, due to the long run of the HPLC and to the acidic conditions. The stability data for molecule **7** are reported in Figure 5 and Table 2. Briefly, after the preparative HPLC step, the chromatographic purity of the collected fraction was assessed via HLPC-DAD and results showed only one peak corresponding to the activated NHS ester derivative **7**. After storing compound **7** at −21 °C for 7 days, HPLC-DAD analysis was performed, and the chromatographic analysis indicated high stability. Indeed, the major peak (89%) corresponded to the NHS ester derivative **7** while the smaller peak corresponded to the acid derivatives **7h** (11%). After 30 days at −21 °C, the amount of **7h** remained largely unchanged (12%). Therefore **7h** was most probably formed due to initial hydrolyzation during the freeze-drying process rather than degradation during storage. After 12 h in water solution at room temperature, as expected and in line with NHS chemistry, the only detected product was the hydrolyzed ester **7h**.

The stability of **7** over a month and in conjugation conditions: DMSO/DMF for solid-phase synthesis and PBS for proteins/antibodies conjugation, were also verified with excellent results and showing excellent stability over the studied period (Table 2). Considering the promising stability results of this compound we decided to start a pilot bio-conjugation with a peptide that binds the GLP-1 receptor, to evaluate the feasibility of the reaction and the actual chelation properties of the bioconjugate for gallium-68.

### 3.2. Design and Synthesis of GLP-1-Peptide–THP

To assess the feasibility of molecule **7** to effectively form amide bonds under mild conditions without any activation agent, a potential PET radiotracer-a peptide targeting the GLP-1 receptor was designed and conjugated to the newly synthesized NHS-THP **7**. As already reported, the GLP-1R has been shown to be expressed in high density and high incidence in different types of cancers derived from endocrine, neuroendocrine, and embryonic origins (e.g., insulinoma). A PET imaging tracer can be particularly useful for the detection of primary and small metastatic lesions and it can generally support the diagnosis, staging and treatment monitoring of patients with GLP-1R positive malignancies.

**Figure 6:**
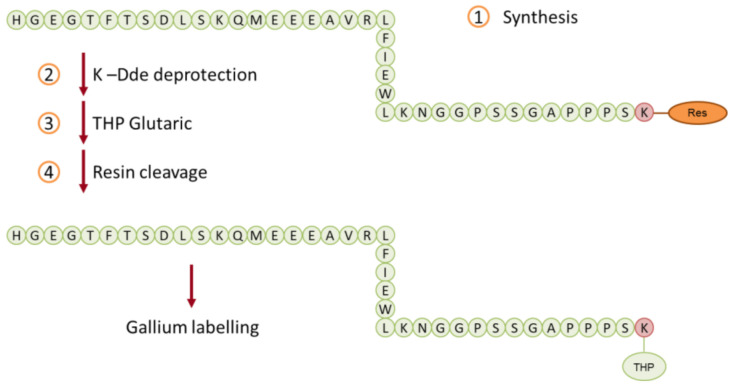
Schematic synthesis of GLP-1-peptide-THP **8**.

As targeting peptide, the sequence of exendin-4 was chosen. It was already reported that the peptide can be opportunely modified by the insertion of a terminal linker amino acid (e.g., lysine or cysteine) without losing its activity/affinity toward the GLP-1 receptor (Figure 3) and then used in PET imaging making it an active radiopharmaceutical agent. For this study, it was decided to conjugate the same peptide (HGEGTFTSDLSKQMEEEAVRLFIEWLKNGGPSSGAPPPS) with a terminal lysine, then to a hexadentate 3,4-hydroxypyridinone ligand (NHS-THP **7**) exploiting NHS chemistry (Figure 6). The peptide was manually assembled using Fmoc chemistry (Figure 6 step 1). After the linear sequence was obtained and after the removal of the Dde protecting group (Figure 6, step 2), the **7** was coupled to the peptide (Figure 6, step 3). After the coupling of **7**, the peptide was removed from the solid support (Figure 6, step 4).

**Figure 7:**
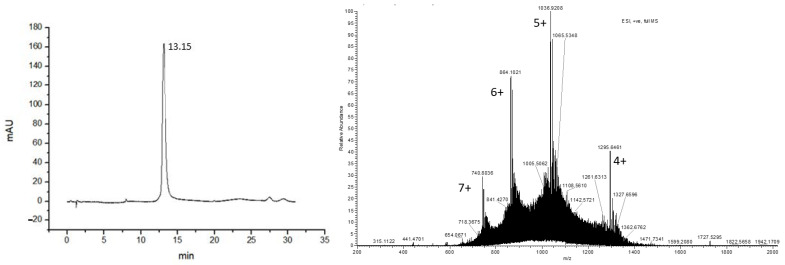
**Left**: HPLC chromatogram of GLP-1-peptide-THP (**8**); **right**: HRMS of GLP-1-peptide-THP (**8**).

Once the synthesis of the GLP-1-peptide-THP (**8**) was completed, the compound was purified via preparative HPLC/UV. The isolated fractions were further analyzed by analytical HPLC-DAD, and the pure fractions (Figure 7) were analyzed by mass spectrometry (HRMS, ESI^+^). The identification of the final product was confirmed by the mass spectrometry experiments as highlighted in Figure 7, where the [M + 4H]^4+^, [M + 5H]^5+^, [M + 6H]^6+^, [M + 7H]^7+^ of GLP-1-peptide-THP conjugate are shown.

### 3.3. ^68^Ga Radiolabeling of GLP-1-Peptide-THP: Characterization by Radio HPLC and ITLC

To evaluate the chelating ability of the synthesized GLP-1-peptide-THP (**8**) for ^68^Ga, instant thin layer chromatography (ITLC) analyses and radio HPLC analyses were used. Two different methods were used in the ITLC analyses. The different methods were named “acetate method” and “citrate method” and the results are reported in Table 3 and in Figures S6–S9. As expected, the acetate method was unable to identify the chelating product, as under these conditions the [^68^Ga]Ga-GLP-1-peptide-THP (**9**) has a retention factor of 0 which is the same as that of the free gallium-68 acetate (Figures S6 and S8). The citrate method, however, could clearly separate free gallium-68 from the radiolabeled peptide: In this system, the labeled peptide **9** has a retention factor of 0 and free gallium-68 has a retention factor of 0.8–1 (Figures S7 and S9). This result suggests that the peptide is chelating gallium-68. Similar results were obtained with the HPLC analyses, where a reverse phase (C18) column was used: the results reported in Table 4 confirm the formation of **9**. In the HPLC analyses, the reverse phase system shows retention times of 1.9 and 13.7–13.9 min for unbound ^68^Ga and of **9**, respectively. Each analysis gave a single peak in the HPLC radiochromatograms (See Supplementary Material, Figures S10 and S11), and under these conditions, the radiochemical yield for all these products was >95% (determined by ITLC).

### 3.4. Synthesis of MY-1502-6-51-THP

Samples of the isolated sdAb conjugate 10, prepared with 20-fold and 40-fold molar excess of 7 were analyzed by high-resolution mass spectrometry in full-scan and in positive electrospray ionization mode. No major difference was observed in the mass spectra of the two conjugates: a peak corresponding to the native sdAb was observed (see Figures S12–S14), however, in the conjugated sample, a peak corresponding to the 1:1 conjugate 10 was also detectable, demonstrating qualitatively the successful conjugation due to amide bond formation between the sdAb and the THP-NHS ligand under mild conditions. Conjugates with more than one THP chelator per sdAb were not identified. Due to their similar molecular weight, it was not possible to separate sdAb from sdAb-THP conjugates by SEC-HPLC in this preliminary study.

### 3.5. ^68^Ga Radiolabeling of MY-1502-6-51-THP

The sdAb conjugate 10 was radiolabeled with ^68^Ga (Figures S15–S18). Without optimizing radiolabeling conditions, radiolabeling yields were >90% after a 5-min incubation at room temperature: 91% with the conjugate prepared in 20-fold excess of 7 (Figures S15 and S16) and 92% with the conjugate prepared in 40-fold excess of 7, no difference was observed between the two conjugates (Figure S17). The concentration of the sdAb-THP conjugate in the reaction mixture was 15 µM, this is in line with the 7 µM concentration in other THP-conjugates [17,19]. The radiolabeling yield of the sdAb incubated with the non-functionalized THP 5 was 0% confirming that the association of ^68^Ga with 10 was THP-mediated (Figure S18). Results are summarized in Table 5 and Figures S15–S18.

## 4. Conclusions and Perspective

In this paper, we developed a versatile NHS-THP metal chelator that could be conjugated virtually to any targeting molecule that contains a primary amine, exploiting the advantages of the NHS chemistry and THP for gallium radiochemistry. To demonstrate its properties, the obtained molecule was conjugated to a GLP-1R targeting peptide and the bioconjugate was studied for its gallium chelating capabilities showing >95% radiochemical yield. Even if the proposed GLP-1R targeting peptide (**8**) synthesis exploits a solid-phase approach to guarantee a site-specific conjugation of the generated NHS-THP (**7**) to Lys^40^, the same NHS-THP can enable a facile route for the preparation of other protein conjugates containing a tris(hydroxypyridinone) chelator under mild conditions. Particularly the developed NHS-THP (**7**) chelator can be used for simple, efficient labeling of ^67^Ga/^68^Ga biomolecules under suitable conditions for peptides and proteins in aqueous phase and with minimal requirements of synthetic skills. This would greatly increase ^67^Ga/^68^Ga access to hospitals that lack expertise or facilities in preparation of kit-based radiopharmaceuticals. Thus, such kit-based technologies have the potential to expand the use of the ^68^Ga generator for the benefit of more hospitals and patients. The combination of targeted imaging agents with PET allows a non-invasive diagnosis of particular cancers with accurate delineation of the disease staging. Moreover, the uptake kinetics, the quantification of the target receptor expression, and the pre-therapeutic dosimetry may allow a more effective selection of the treatments and planning, as well as monitoring response to the therapy and early detection of recurrent disease resulting in personalized medicine and radiotheranostics. The main aims of individualized patient management strategies are to optimize the treatments and to minimize risks and toxicity as well as reduce cost and patient distress. Clinical studies with different ^68^Ga-based imaging agents demonstrated the significance of individualized patient management [47,48,49].

It is believed that, in future clinical studies, noninvasive PET imaging with radiolabeled tracers such as the synthesized GLP-1-peptide-THP may support the diagnosis of particular types of cancers with overexpression of GLP-1R. Moreover, the new developed NHS-THP represents a new way to easily conjugate the THP moiety to different targeting molecules (e.g., antibodies, anticalins, or peptides) using either solid-phase approach such as in this study, or a solution (non-targeted) approach, paving the way to the synthesis of different products with all the advantages of the THP chemistry. This approach was evaluated in a preliminary set of experiments with a THP conjugated sdAb and we confirmed that the conjugate quantitatively radiolabeled with ^68^Ga at micromolar concentrations.

## Figures and Tables

**Table 1:** Method 1 for radio HPLC: A = water + 0.05% trifluoroacetic acid. B = acetonitrile + 0.05% trifluoroacetic acid. Flow rate: 1 mL/min.

Time/Min	Solvent%	
	A	B
0	95	5
5	95	5
20	5	95
25	5	95
25.1	95	5
30	95	5

**Table 2:** Stability tests (based on HPLC peak area ratios) of **7** (refer to Figure 5), in DMSO/DMF, PBS and over one month of dried compound.

After the Prep HPLC		After 12 h in Water Solution at RT		After 7 Days at −21 °C, Dried Compound	
**NHS ester (7)**	**acid der. (7h)**	**NHS ester (7)**	**acid der. (7h)**	**NHS ester (7)**	**acid der. (7h)**
100	0	0	100	89	11
**1h DMSO/DMF**		**1h PBS**		**After 30 days at −21 °C, dried compound**	
**NHS ester (7)**	**acid der. (7h)**	**NHS ester (7)**	**acid der. (7h)**	**NHS ester (7)**	**acid der. (7h)**
97	3	91	9	88	12

**Table 3:** Results of the ITLC analysis.

	Acetate Method R_f_	Citrate Method R_f_
^68^Ga unbound	0	0.8–1
[^68^Ga]Ga-GLP-1-peptide-THP (**9**)	0	0

**Table 4:** Results of the HPLC analyses.

	Reverse Phase R_t_ (min)
^68^Ga unbound	1.9
[^68^Ga]Ga-GLP-1-peptide-THP (**9**)	13.7–13.9

**Table 5:** Results of HPLC analysis of ^68^Ga-labeled sdAb conjugates and corresponding controls.

	Radiochromatogram R_t_ (min)	UV R_t_ (min)
^68^Ga unbound	10.7–10.8	-
[^68^Ga]Ga-MY-1502-6-51-THP (**11**)	6.7–6.8	6.2–6.4

## Data Availability

The data presented in this study are available on request from the corresponding author.

## Supplementary Materials

The following are available online at https://www.mdpi.com/article/10.3390/biomedicines9040367/s1, Figure S1: ESI^+^ MS spectra of deprotected THP (5), Figure S2: ^1^H NMR of deprotected THP (5), Figure S3: ^13^C NMR of deprotected THP (5), Figure S4: ESI^+^ MS spectra of NHS-THP-glutaric (7), Figure S5: ^1^H NMR of NHS-THP-glutaric (7), Figure 6S: ITLC acetate unbound ^68^Ga, Figure S7: ITLC citrate unbound ^68^Ga, Figure S8: ITLC acetate [^68^Ga]Ga-GLP-1-peptide-THP (9), Figure S9: ITLC citrate [^68^Ga]Ga-GLP-1-peptide-THP (9), Figure S10: Reverse phase HPLC unbound gallium-68, Figure S11: Reverse phase HPLC [^68^Ga]Ga-GLP-1-peptide-THP (9), Figure S12: ESI+ mass spectrum of MY-1502-6-51, Figure S13: ESI+ mass spectrum of MY-1502-6-51-THP (10) (20-fold molar excess of 7), Figure S14: ESI+ mass spectrum of MY-1502-6-51-THP (10) (40-fold molar excess of 7), Figure S15: HPLC UV chromatogram (Method 2) of MY-1502-6-51-THP (10) (20-fold molar excess of 7), Figure S16: HPLC radiochromatogram (Method 2) of ^68^Ga-MY-1502-6-51-THP (11) (20-fold molar excess of 7), Figure S17: HPLC radiochromatogram (Method 2) of ^68^Ga-MY-1502-6-51-THP (11) (40-fold molar excess of 7), Figure S18: HPLC radiochromatogram (Method 2) and corresponding UV chromatograms (bottom) of ^68^Ga labeled MY-1502-6-51 (20-fold molar excess of 5).

## References

[1] Blower P.J. (2015). A nuclear chocolate box: The periodic table of nuclear medicine. Dalton Trans..

[2] Dansereau R.N., Line B.R. (1996). Clinical production of pharmaceutical grade technetium-99m dextran 70 for lymphoscintigraphy. J. Nucl. Med..

[3] Chomet M., Provost C., Vega V., Prignon A., Talbot J., Nataf V. (2016). Transfer of a radiolabelling process with gallium-68 from a manual method to a remote controlled method for clinical applications: The example of NODAGA-RGDfK. Eur. J. Nucl. Med. Mol. I.

[4] Banerjee S.R., Pomper M.G. (2013). Clinical applications of Gallium-68. Appl. Radiat. Isot..

[5] Ebenhan T., Vorster M., Marjanovic-Painter B., Wagener J., Suthiram J., Modiselle M., Mokaleng B., Zeevaart J.R., Sathekge M. (2015). Development of a Single Vial Kit Solution for Radiolabeling of Ga-68-DKFZ-PSMA-11 and Its Performance in Prostate Cancer Patients. Molecules.

[6] Deutsch E. (1993). Clinical Pet—Its Time Has Come. J. Nucl. Med..

[7] Wagner H.N. (1991). Clinical Pet—Its Time Has Come. J. Nucl. Med..

[8] Benesova M., Schafer M., Bauder-Wust U., Afshar-Oromieh A., Kratochwil C., Mier W., Haberkorn U., Kopka K., Eder M. (2015). Preclinical Evaluation of a Tailor-Made DOTA-Conjugated PSMA Inhibitor with Optimized Linker Moiety for Imaging and Endoradiotherapy of Prostate Cancer. J. Nucl. Med..

[9] Notni J., Pohle K., Wester H.J. (2012). Comparative gallium-68 labeling of TRAP-, NOTA-, and DOTA-peptides: Practical consequences for the future of gallium-68-PET. EJNMMI Res..

[10] Farkas E., Nagel J., Waldron B.P., Parker D., Toth I., Brucher E., Rosch F., Baranyai Z. (2017). Equilibrium, Kinetic and Structural Properties of Gallium(III) and Some Divalent Metal Complexes Formed with the New DATA(m) and DATA(5m) Ligands. Chem. Eur. J..

[11] Ramogida C.F., Schindler D., Schneider C., Tan Y.L.K., Huh S., Ferreira C.L., Adam M.J., Orvig C. (2016). Synthesis and characterization of lipophilic cationic Ga(III) complexes based on the H(2)CHXdedpa and H(2)dedpa ligands and their Ga-67/68 radiolabeling studies. RSC Adv..

[12] Ramogida C.F., Pan J.H., Ferreira C.L., Patrick B.O., Rebullar K., Yapp D.T.T., Lin K.S., Adam M.J., Orvig C. (2015). Nitroimidazole-Containing H(2)dedpa and H(2)CHXdedpa Derivatives as Potential PET Imaging Agents of Hypoxia with Ga-68. Inorg. Chem..

[13] Seemann J., Waldron B.P., Roesch F., Parker D. (2015). Approaching ‘Kit-Type’ Labelling with Ga-68: The DATA Chelators. ChemMedChem.

[14] Cilibrizzi A., Abbate V., Chen Y.-L., Ma Y., Zhou T., Hider R.C. (2018). Hydroxypyridinone Journey into Metal Chelation. Chem. Rev..

[15] Berry D.J., Ma Y., Ballinger J.R., Tavare R., Koers A., Sunassee K., Zhou T., Nawaz S., Mullen G.E., Hider R.C. (2011). Efficient bifunctional gallium-68 chelators for positron emission tomography: Tris(hydroxypyridinone) ligands. Chem. Commun..

[16] Ma M.T., Cullinane C., Imberti C., Baguna Torres J., Terry S.Y., Roselt P., Hicks R.J., Blower P.J. (2016). New Tris(hydroxypyridinone) Bifunctional Chelators Containing Isothiocyanate Groups Provide a Versatile Platform for Rapid One-Step Labeling and PET Imaging with ^68^Ga^3+^. Bioconjug. Chem..

[17] Young J.D., Abbate V., Imberti C., Meszaros L.K., Ma M.T., Terry S.Y.A., Hider R.C., Mullen G.E., Blower P.J. (2017). (68)Ga-THP-PSMA: A PET Imaging Agent for Prostate Cancer Offering Rapid, Room-Temperature, 1-Step Kit-Based Radiolabeling. J. Nucl. Med..

[18] Keeling G.P., Sherin B., Kim J., San Juan B., Grus T., Eykyn T.R., Rösch F., Smith G.E., Blower P.J., Terry S.Y.A. (2020). [68Ga]Ga-THP-Pam: A Bisphosphonate PET Tracer with Facile Radiolabeling and Broad Calcium Mineral Affinity. Bioconjug. Chem..

[19] Hofman M.S., Eu P., Jackson P., Hong E., Binns D., Iravani A., Murphy D., Mitchell C., Siva S., Hicks R.J. (2018). Cold Kit for Prostate-Specific Membrane Antigen (PSMA) PET Imaging: Phase 1 Study of (68)Ga-Tris(Hydroxypyridinone)-PSMA PET/CT in Patients with Prostate Cancer. J. Nucl. Med..

[20] Eisenhut M., Lehmann W.D., Becker W., Behr T., Elser H., Strittmatter W., Steinstrasser A., Baum R.P., Valerius T., Repp R. (1996). Bifunctional NHS-BAT ester for antibody conjugation and stable technetium-99m labeling: Conjugation chemistry, immunoreactivity and kit formulation. J. Nucl. Med..

[21] Cusnir R., Imberti C., Hider R.C., Blower P.J., Ma M.T. (2017). Hydroxypyridinone Chelators: From Iron Scavenging to Radiopharmaceuticals for PET Imaging with Gallium-68. Int. J. Mol. Sci..

[22] Nawaz S., Mullen G.E.D., Sunassee K., Bordoloi J., Blower P.J., Ballinger J.R. (2017). Simple, mild, one-step labelling of proteins with gallium-68 using a tris(hydroxypyridinone) bifunctional chelator: A (68)Ga-THP-scFv targeting the prostate-specific membrane antigen. EJNMMI Res..

[23] Aroda V.R. (2018). A review of GLP-1 receptor agonists: Evolution and advancement, through the lens of randomised controlled trials. Diabetes Obes. Metab..

[24] Gallwitz B. (2011). GLP-1 agonists and dipeptidyl-peptidase IV inhibitors. Handb. Exp. Pharmacol..

[25] Lugari R., Dei Cas A., Ugolotti D., Barilli A.L., Camellini C., Ganzerla G.C., Luciani A., Salerni B., Mittenperger F., Nodari S. (2004). Glucagon-like peptide 1 (GLP-1) secretion and plasma dipeptidyl peptidase IV (DPP-IV) activity in morbidly obese patients undergoing biliopancreatic diversion. Horm. Metab. Res..

[26] Gallwitz B., Ropeter T., Morys-Wortmann C., Mentlein R., Siegel E.G., Schmidt W.E. (2000). GLP-1-analogues resistant to degradation by dipeptidyl-peptidase IV in vitro. Regul. Pept..

[27] Nomiyama T., Kawanami T., Irie S., Hamaguchi Y., Terawaki Y., Murase K., Tsutsumi Y., Nagaishi R., Tanabe M., Morinaga H. (2014). Exendin-4, a GLP-1 receptor agonist, attenuates prostate cancer growth. Diabetes.

[28] Ryder R.E. (2013). The potential risks of pancreatitis and pancreatic cancer with GLP-1-based therapies are far outweighed by the proven and potential (cardiovascular) benefits. Diabet. Med..

[29] Mehrabi A., Fischer L., Hafezi M., Dirlewanger A., Grenacher L., Diener M.K., Fonouni H., Golriz M., Garoussi C., Fard N. (2014). A systematic review of localization, surgical treatment options, and outcome of insulinoma. Pancreas.

[30] Trujillo J.M., Nuffer W., Ellis S.L. (2015). GLP-1 receptor agonists: A review of head-to-head clinical studies. Ther. Adv. Endocrinol. Metab..

[31] Wong M.C., Wang H.H., Kwan M.W., Zhang D.D., Liu K.Q., Chan S.W., Fan C.K., Fong B.C., Li S.T., Griffiths S.M. (2014). Comparative effectiveness of dipeptidyl peptidase-4 (DPP-4) inhibitors and human glucagon-like peptide-1 (GLP-1) analogue as add-on therapies to sulphonylurea among diabetes patients in the Asia-Pacific region: A systematic review. PLoS ONE.

[32] Derosa G., Maffioli P. (2012). GLP-1 agonists exenatide and liraglutide: A review about their safety and efficacy. Curr. Clin. Pharmacol..

[33] Wild D., Wicki A., Mansi R., Behe M., Keil B., Bernhardt P., Christofori G., Ell P.J., Macke H.R. (2010). Exendin-4-based radiopharmaceuticals for glucagonlike peptide-1 receptor PET/CT and SPECT/CT. J. Nucl. Med..

[34] Brom M., Joosten L., Oyen W.J., Gotthardt M., Boerman O.C. (2012). Radiolabelled GLP-1 analogues for in vivo targeting of insulinomas. Contrast Media Mol. Imaging.

[35] Pach D., Sowa-Staszczak A., Jabrocka-Hybel A., Stefanska A., Tomaszuk M., Mikolajczak R., Janota B., Trofimiuk-Muldner M., Przybylik-Mazurek E., Hubalewska-Dydejczyk A. (2013). Glucagon-Like Peptide-1 Receptor Imaging with [Lys (40) (Ahx-HYNIC- (99 m) Tc/EDDA)NH 2 ]-Exendin-4 for the Diagnosis of Recurrence or Dissemination of Medullary Thyroid Cancer: A Preliminary Report. Int. J. Endocrinol..

[36] Sowa-Staszczak A., Pach D., Mikolajczak R., Macke H., Jabrocka-Hybel A., Stefanska A., Tomaszuk M., Janota B., Gilis-Januszewska A., Malecki M. (2013). Glucagon-like peptide-1 receptor imaging with [Lys40(Ahx-HYNIC- 99mTc/EDDA)NH2]-exendin-4 for the detection of insulinoma. Eur. J. Nucl. Med. Mol. Imaging.

[37] Kiesewetter D.O., Gao H., Ma Y., Niu G., Quan Q., Guo N., Chen X. (2012). 18F-radiolabeled analogs of exendin-4 for PET imaging of GLP-1 in insulinoma. Eur. J. Nucl. Med. Mol. Imaging.

[38] Hubalewska-Dydejczyk A., Sowa-Staszczak A., Tomaszuk M., Stefanska A. (2015). GLP-1 and exendin-4 for imaging endocrine pancreas. A review. Labelled glucagon-like peptide-1 analogues: Past, present and future. Q. J. Nucl. Med. Mol. Imaging.

[39] Muyldermans S. (2013). Nanobodies: Natural single-domain antibodies. Annu. Rev. Biochem..

[40] Xing Y., Chand G., Liu C., Cook G.J.R., O’Doherty J., Zhao L., Wong N.C.L., Meszaros L.K., Ting H.H., Zhao J. (2019). Early Phase I Study of a (99 m)Tc-Labeled Anti-Programmed Death Ligand-1 (PD-L1) Single-Domain Antibody in SPECT/CT Assessment of PD-L1 Expression in Non-Small Cell Lung Cancer. J. Nucl. Med..

[41] Keyaerts M., Xavier C., Heemskerk J., Devoogdt N., Everaert H., Ackaert C., Vanhoeij M., Duhoux F.P., Gevaert T., Simon P. (2016). Phase I Study of 68Ga-HER2-Nanobody for PET/CT Assessment of HER2 Expression in Breast Carcinoma. J. Nucl. Med..

[42] Huyvetter M., De Vos J., Caveliers V., Vaneycken I., Heemskerk J., Duhoux F.P., Fontaine C., Vanhoeij M., Windhorst A.D., van der Aa F. (2020). Phase I trial of ^131^I-GMIB-Anti-HER_2_-VHH_1_, a new promising candidate for HER2-targeted radionuclide therapy in breast cancer patients. J. Nucl. Med..

[43] Castanar L., Poggetto G.D., Colbourne A.A., Morris G.A., Nilsson M. (2018). The GNAT: A new tool for processing NMR data. Magn. Reason. Chem..

[44] Zhou T., Neubert H., Liu D.Y., Liu Z.D., Ma Y.M., Kong X.L., Luo W., Mark S., Hider R.C. (2006). Iron binding dendrimers: A novel approach for the treatment of haemochromatosis. J. Med. Chem..

[45] Van Dongen S.F., Maiuri P., Marie E., Tribet C., Piel M. (2013). Triggering cell adhesion, migration or shape change with a dynamic surface coating. Adv. Mater..

[46] Flakus H.T., Hachula B., Holaj-Krzak J.T. (2015). Long-distance inter-hydrogen bond coupling effects in the polarized IR spectra of succinic acid crystals. Spectrochim. Acta A Mol. Biomol. Spectrosc..

[47] Velikyan I., Sundin A., Eriksson B., Lundqvist H., Sorensen J., Bergstrom M., Langstrom B. (2010). In vivo binding of [68Ga]-DOTATOC to somatostatin receptors in neuroendocrine tumours—Impact of peptide mass. Nucl. Med. Biol..

[48] Velikyan I., Rosenstrom U., Estrada S., Ljungvall I., Haggstrom J., Eriksson O., Antoni G. (2014). Synthesis and preclinical evaluation of 68Ga-labeled collagelin analogs for imaging and quantification of fibrosis. Nucl. Med. Biol..

[49] Selvaraju R.K., Velikyan I., Asplund V., Johansson L., Wu Z., Todorov I., Shively J., Kandeel F., Eriksson B., Korsgren O. (2014). Pre-clinical evaluation of [(68)Ga]Ga-DO3A-VS-Cys(40)-Exendin-4 for imaging of insulinoma. Nucl. Med. Biol..

