# Supplementary material for: NHS-Functionalized THP Derivative for Efficient Synthesis of Kit-Based Precursors for 68Ga Labeled PET Probes
Source: Biomedicines. 2021 Apr 1;9(4):367. doi: 10.3390/biomedicines9040367 (PMC8066796; doi:10.3390/biomedicines9040367)
Supplement: Supplementary file 1 [file biomedicines-09-00367-s001.pdf]

biomedicines

Supplementary materials

NHS-functionalized THP derivative for efficient synthesis of kit-based precursors for ^{68}Ga labelled PET probes

Giuseppe Floresta, George P. Keeling, Siham Memdouh, Levente K. Meszaros Rafael T. M. de Rosales and Vincenzo Abbate

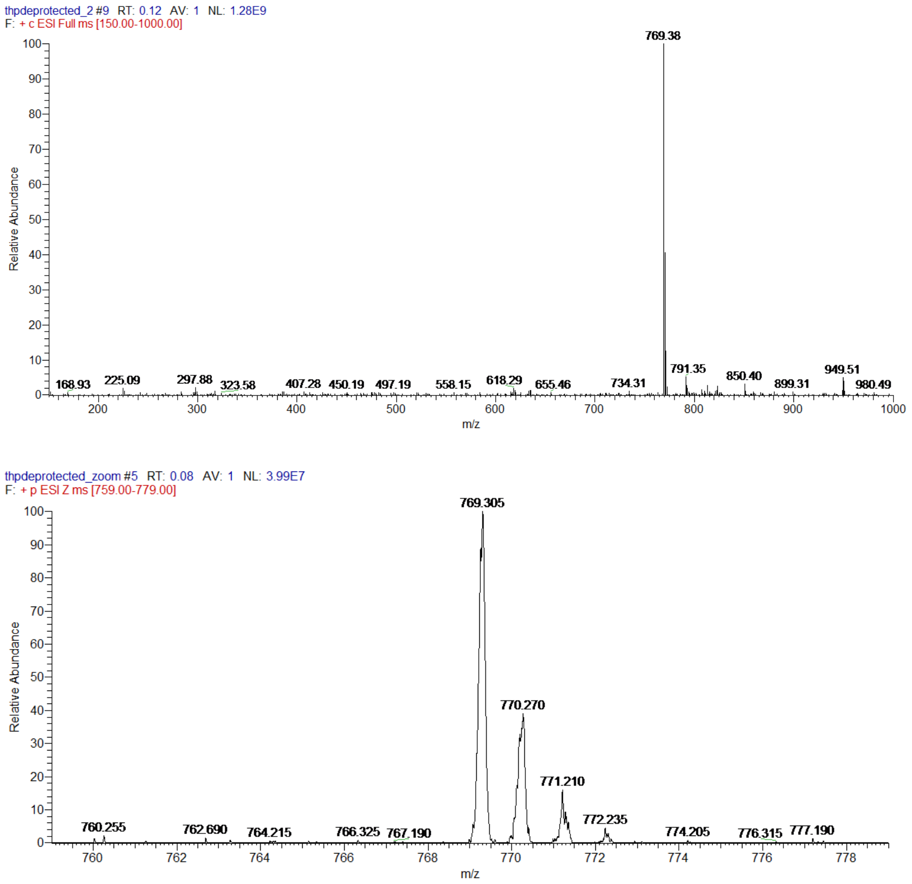

Figure S1. ESI$^+$ MS spectra of deprotected THP (**5**).

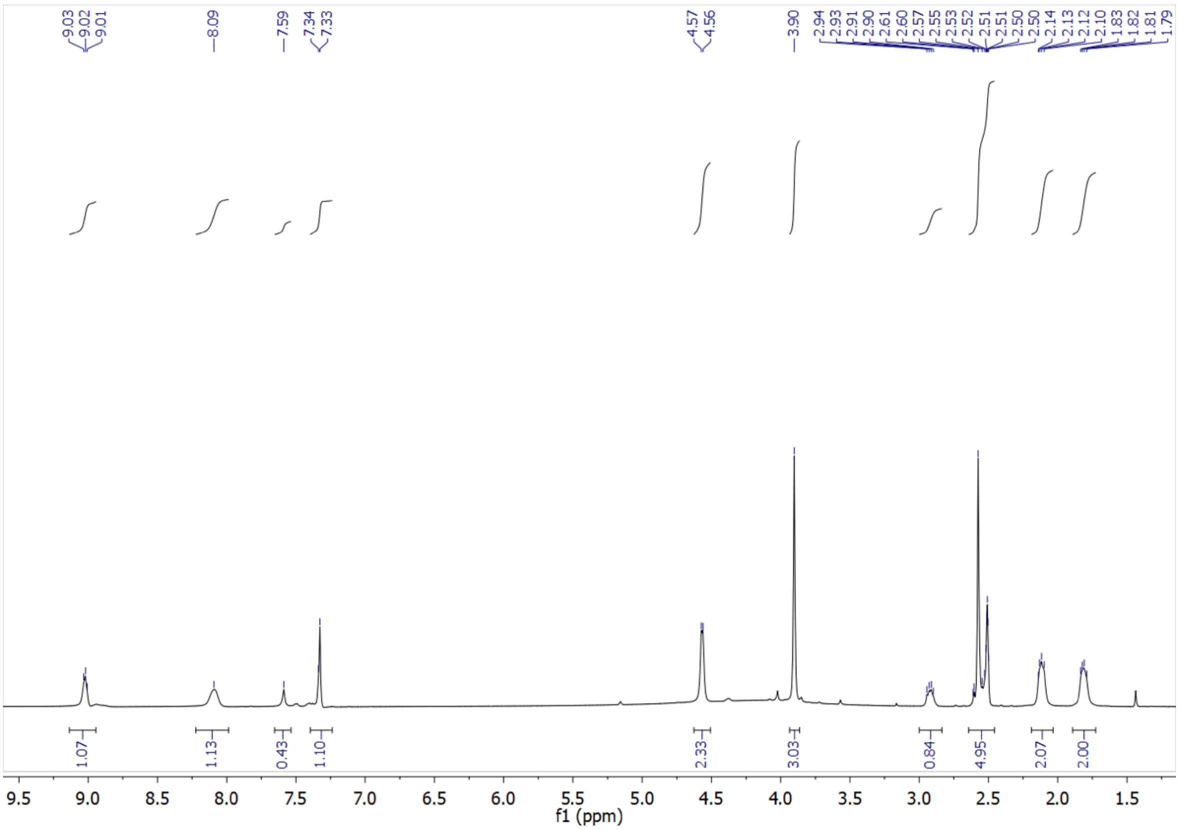

Figure S2. ^{1}H NMR of deprotected THP (**5**).

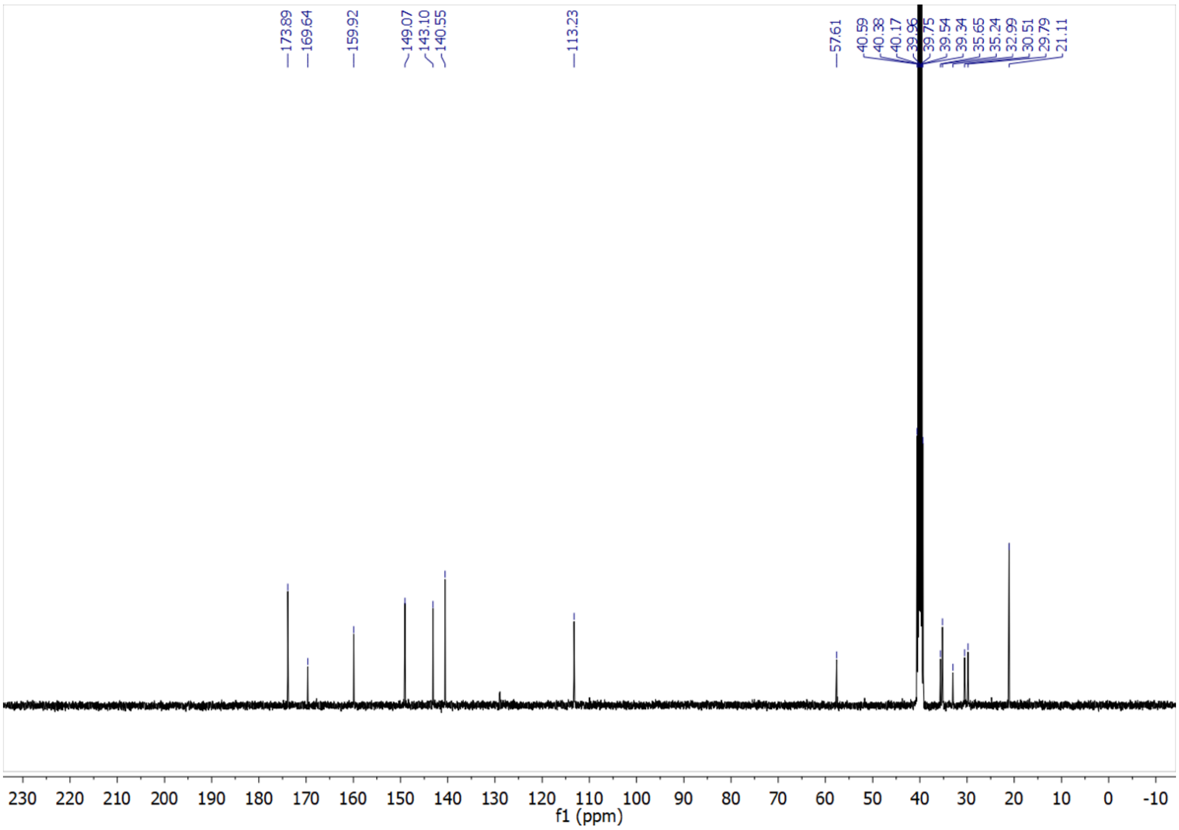

Figure S3. ^{13}C NMR of deprotected THP (**5**).

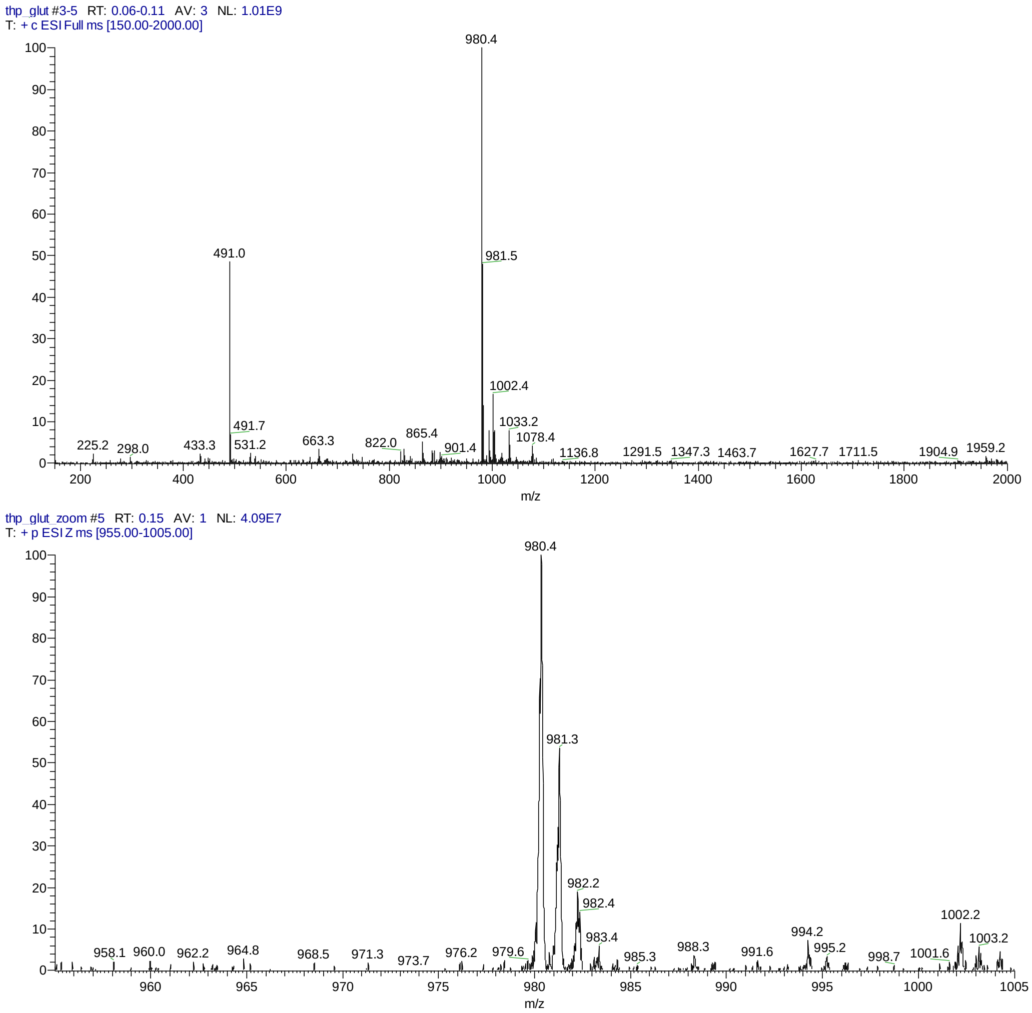

Figure S4. ESI+ MS spectra of NHS-THP-glutaric (**7**).

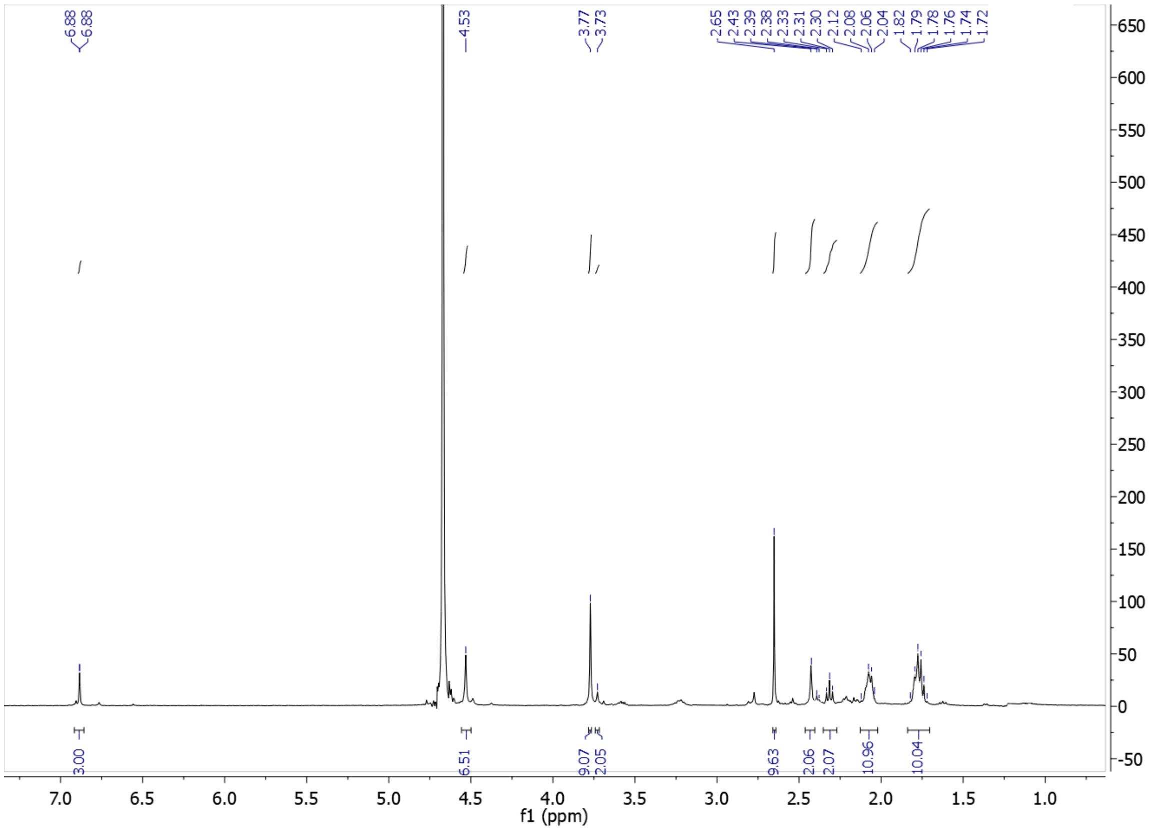

Figure S5. ^{1}H NMR of NHS-THP-glutaric (**7**).

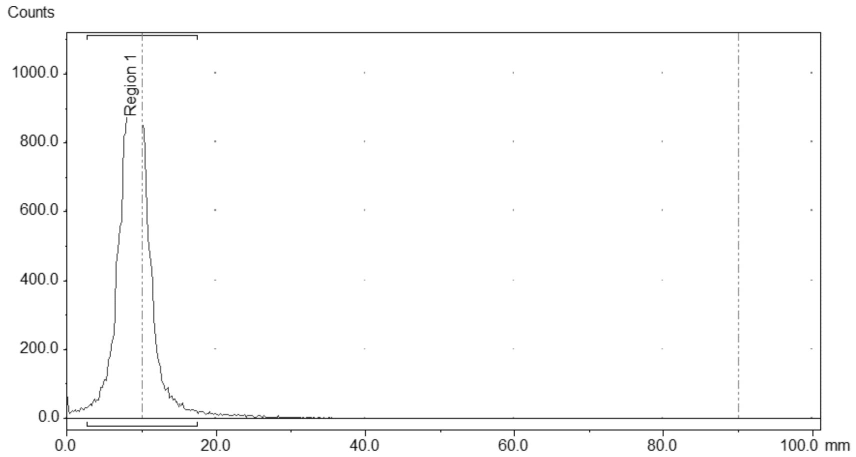

Figure S6. ITLC acetate unbound ^{68}Ga.

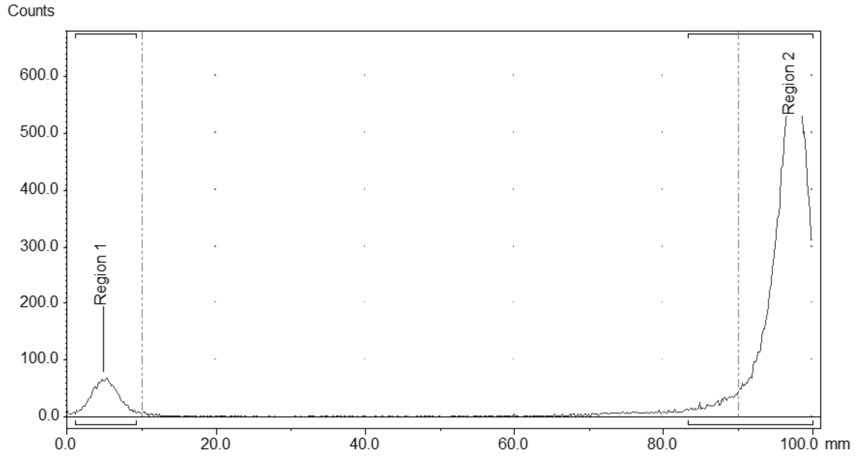

Figure S7. ITLC citrate unbound ^{68}Ga.

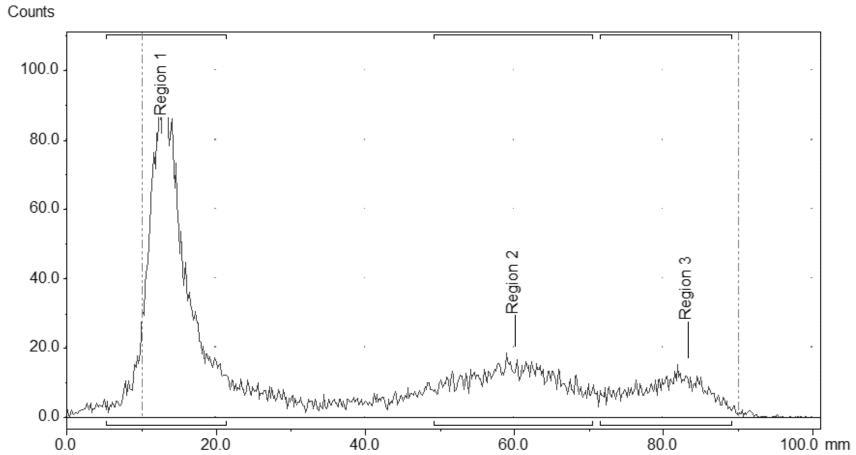

Figure S8. ITLC acetate [^{68}Ga]Ga-GLP-1-peptide-THP (**9**).

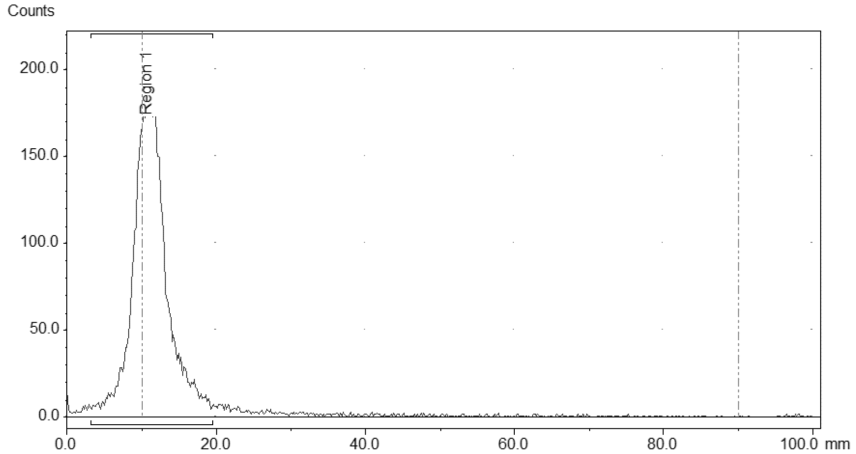

Figure S9. ITLC citrate [^{68}Ga]Ga-GLP-1-peptide-THP (**9**).

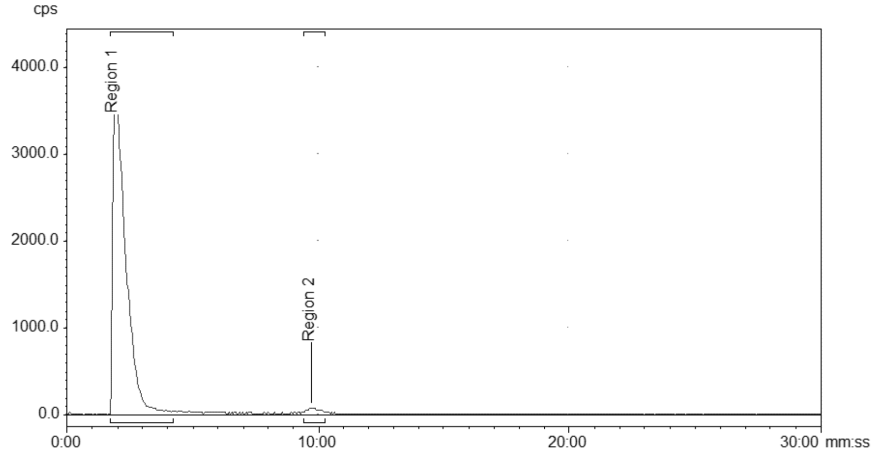

Figure S10. Reverse phase HPLC unbound gallium.

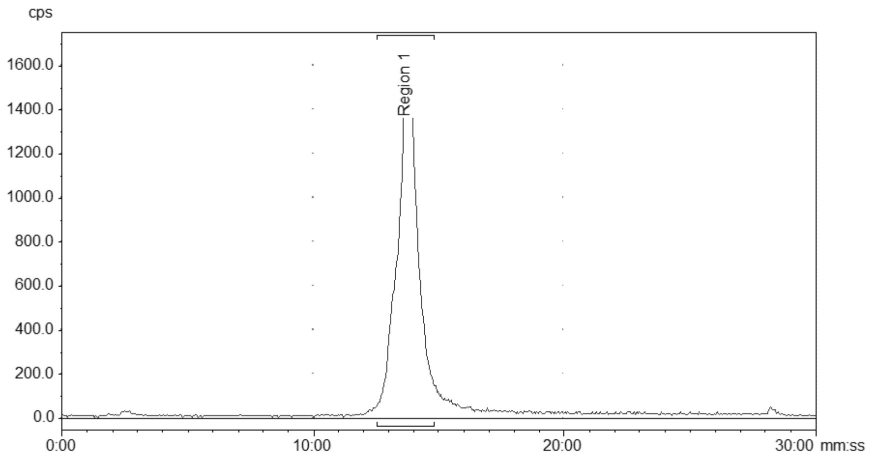

Figure S11. Reverse phase HPLC [^{68}Ga]Ga-GLP-1-peptide-THP (**9**).

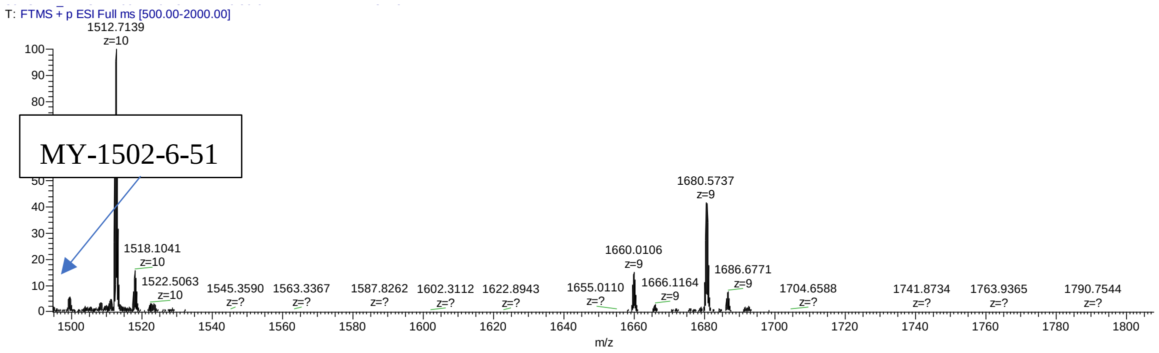

Figure S12.ESI+ mass spectrum of MY-1502-6-51. The peak at m/z 1518 corresponds to the $[M+10H]^{10+}$ of MY-1502-6-51.

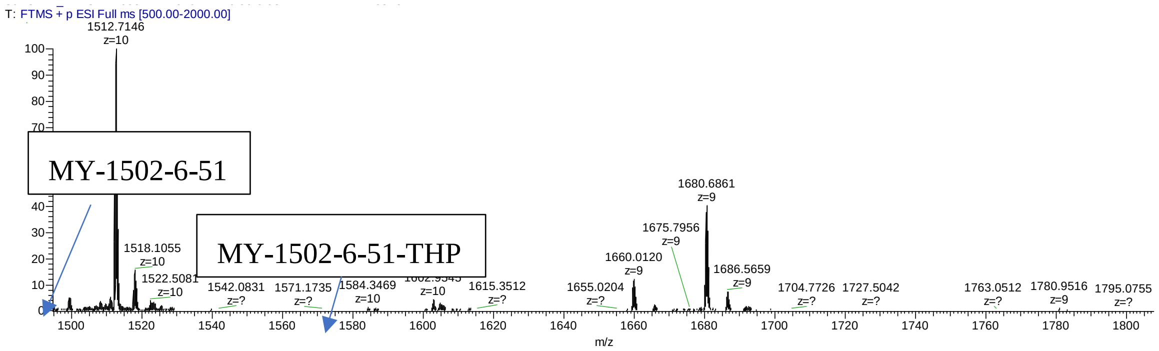

Figure S13. ESI+ mass spectrum of MY-1502-6-51-THP (**10**) (20-fold molar excess of **7**). The peak at m/z 1518 corresponds to the $[M+10H]^{10+}$ of MY-1502-6-51. The peak at 1603 corresponds to $[M+10H]^{0+}$ of 1MY-1502-6-51-THP.

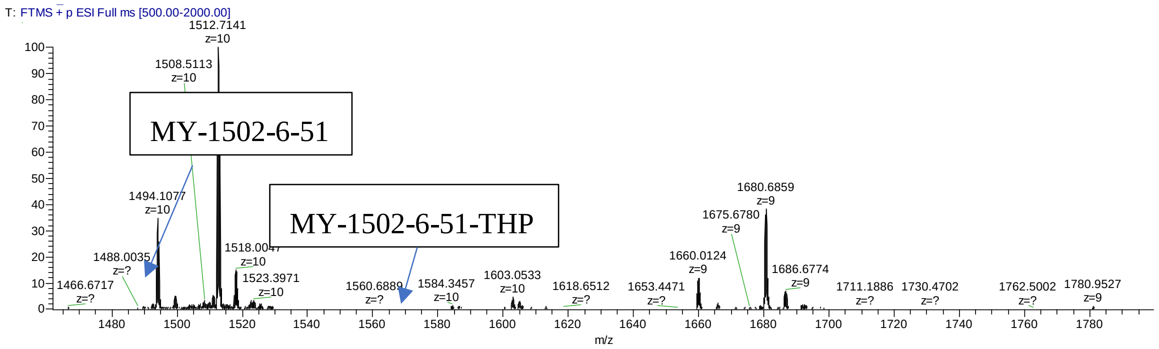

Figure S14. ESI+ mass spectrum of MY-1502-6-51-THP (**10**) (40-fold molar excess of **7**). The peak at m/z 1518 corresponds to the $[M+10H]^{10+}$ of MY-1502-6-51. The peak at 1603 corresponds to $[M+10H]^{10+}$ of MY-1502-6-51-THP.

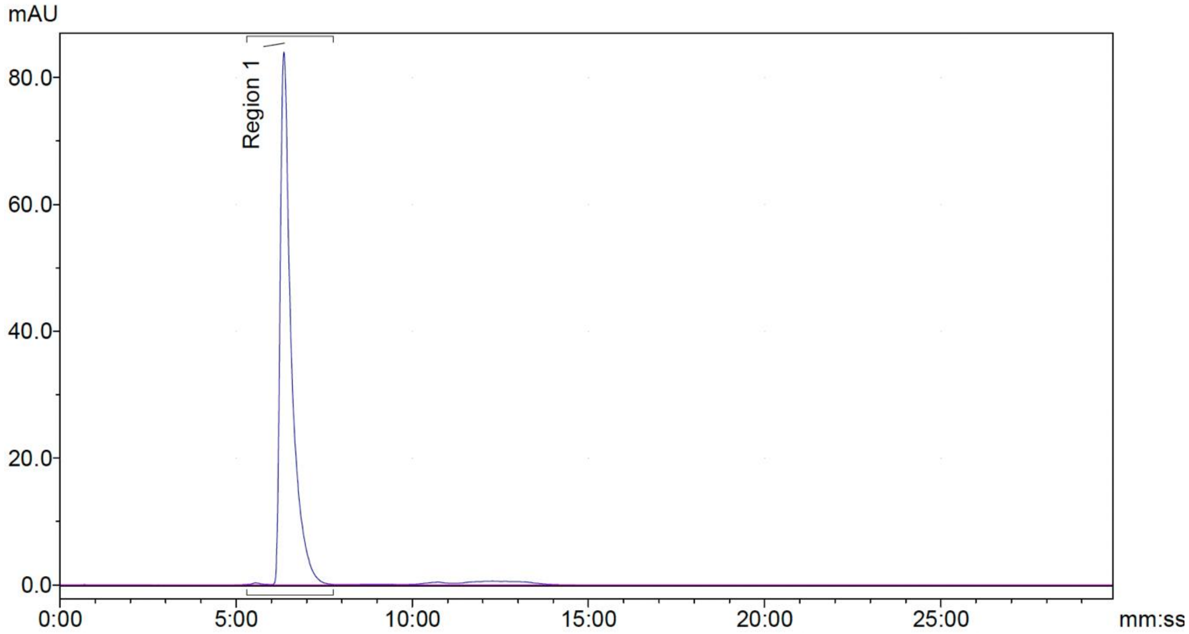

Figure S15. HPLC UV chromatogram (Method 2) of MY-1502-6-51-THP (**10**) (20-fold molar excess of **7**). Region 1 (Rt=6.4 min, 100%) represents MY-1502-6-51-THP. .

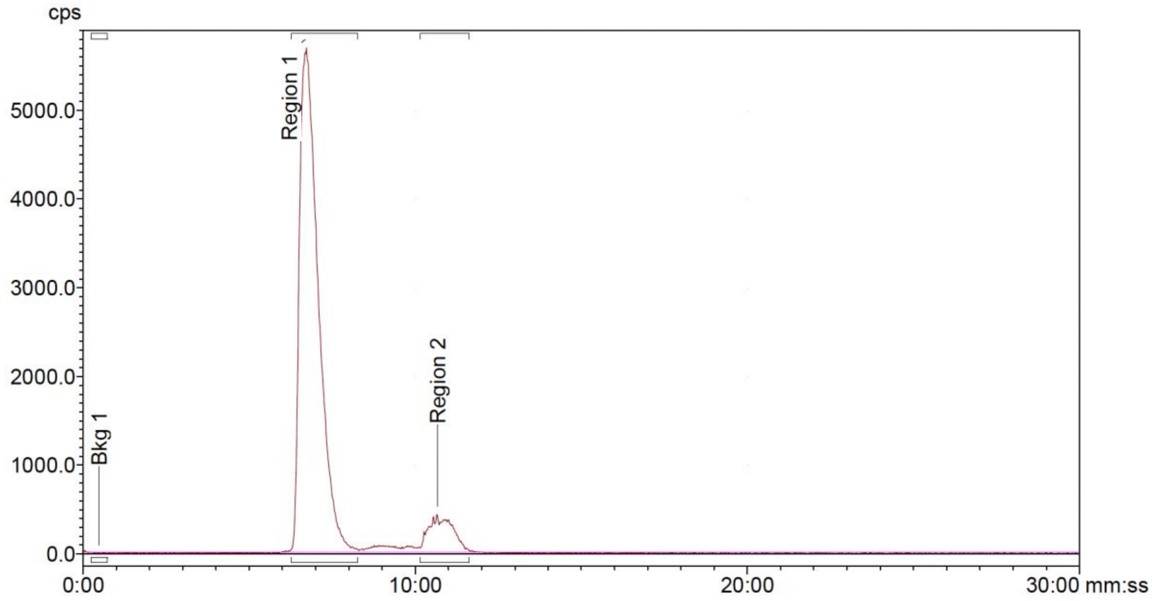

Figure S16. HPLC radiochromatogram (Method 2) of ^{68}Ga-MY-1502-6-51-THP (**11**) (20-fold molar excess of **7**). Region 1 (Rt=6.7 min, 91%) represents ^{68}Ga-MY-1502-6-51-THP, region 2 (Rt=10.7 min, 9%) represents free ^{68}Ga.

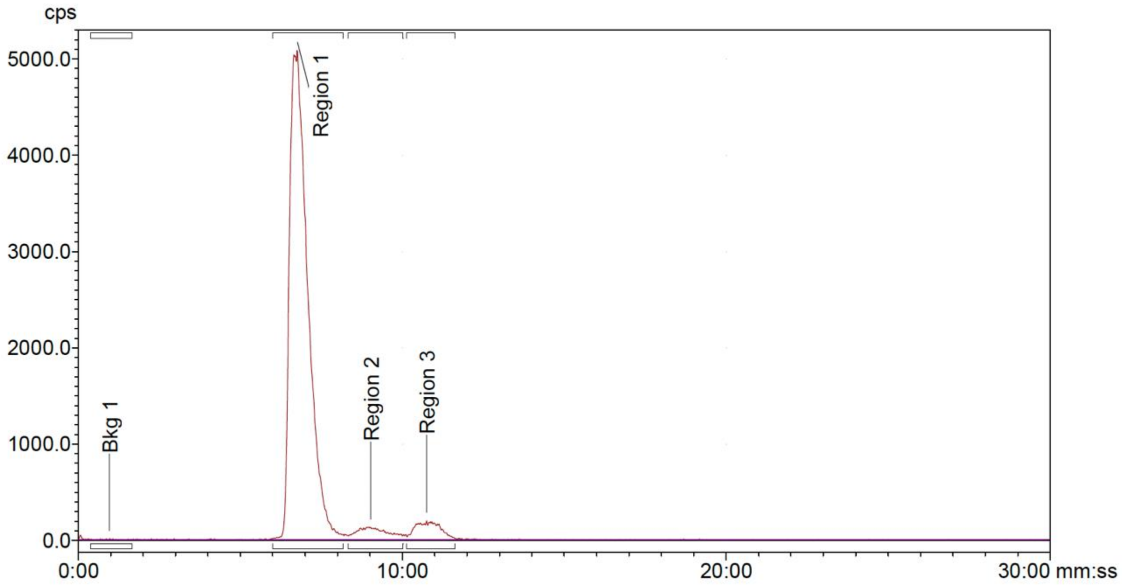

Figure S17. HPLC radiochromatogram (Method 2) of ^{68}Ga-MY-1502-6-51-THP (**11**) (40-fold molar excess of **7**). Region 1 (Rt=6.8 min, 92%) represents ^{68}Ga-MY-1502-6-51-THP, region 2 (Rt=9.0 min, 4%) represents a small ^{68}Ga containing species, potentially ^{68}Ga-7 and region 3 (Rt=10.8 min, 4%) represents free ^{68}Ga.

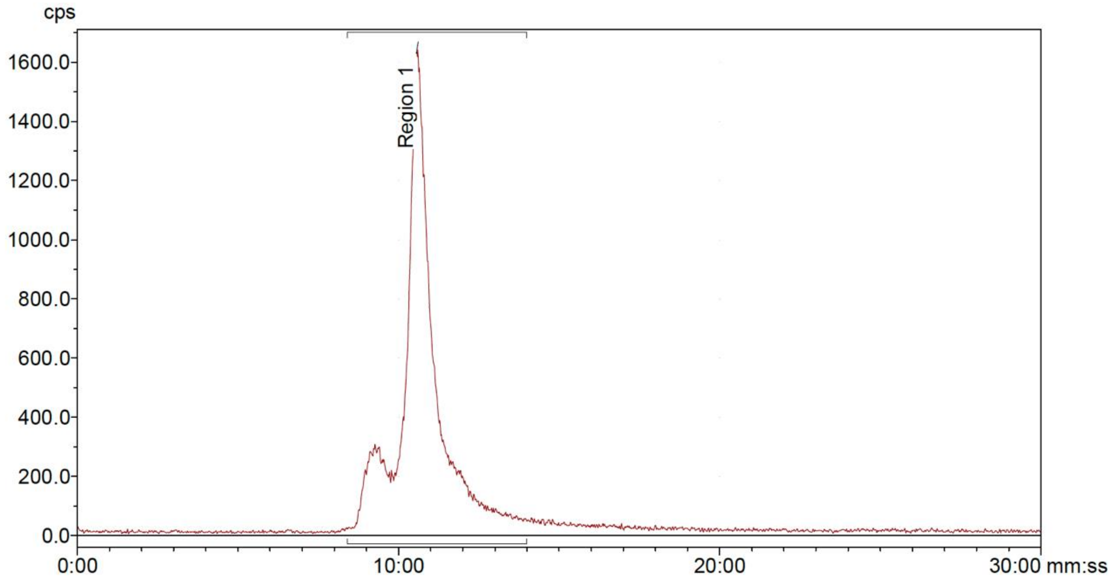

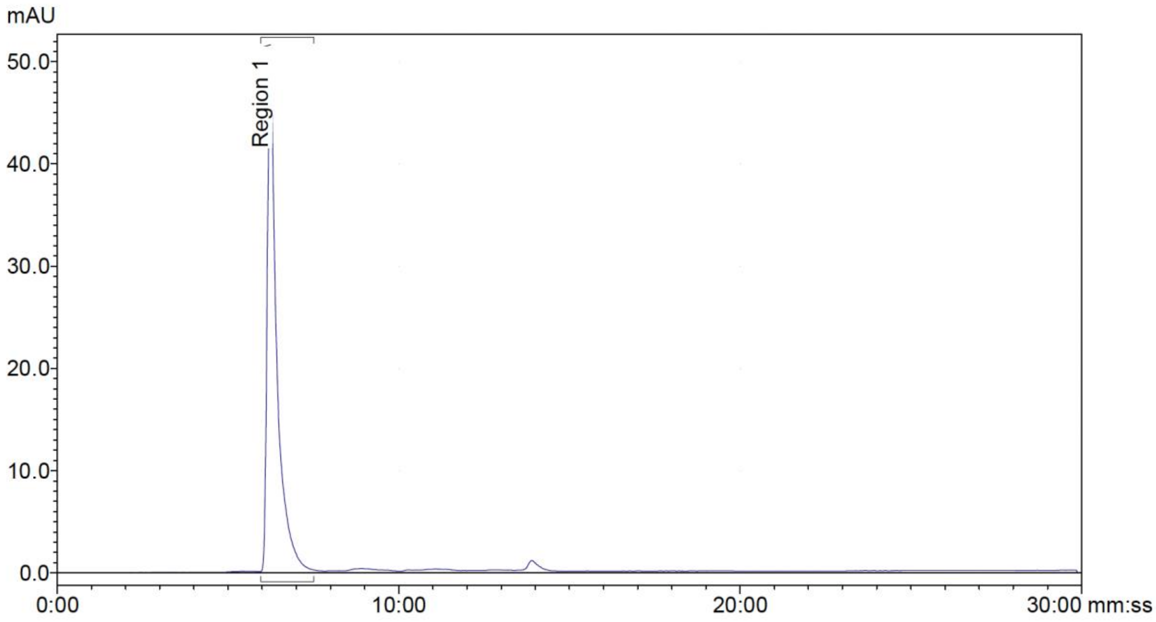

Figure S18.HPLC radiochromatogram (Method 2) and corresponding UV chromatograms (bottom) of ^{68}Ga labeled MY-1502-6-51 (20-fold molar excess of **5**). The peak in the radiochromatogram (Rt=10.8 min) represents free ^{68}Ga with no protein-associated radioactivity present. The UV chromatogram confirmed the presence of MY-1502-6-51 (Rt=6.3 min).
